# Diversity and function of methyl-coenzyme M reductase-encoding archaea in Yellowstone hot springs revealed by metagenomics and mesocosm experiments

**DOI:** 10.1038/s43705-023-00225-9

**Published:** 2023-03-22

**Authors:** Mackenzie M. Lynes, Viola Krukenberg, Zackary J. Jay, Anthony J. Kohtz, Christine A. Gobrogge, Rachel L. Spietz, Roland Hatzenpichler

**Affiliations:** 1 Department of Chemistry and Biochemistry, Center for Biofilm Engineering, and Thermal Biology Institute, Montana State University, Bozeman, MT 59717 USA; 2 Environmental Analytical Lab, Montana State University, Bozeman, MT 59717 USA; 3 Department of Microbiology and Cell Biology, Montana State University, Bozeman, MT 59717 USA

**Keywords:** Environmental microbiology, Archaea

## Abstract

Metagenomic studies on geothermal environments have been central in recent discoveries on the diversity of archaeal methane and alkane metabolism. Here, we investigated methanogenic populations inhabiting terrestrial geothermal features in Yellowstone National Park (YNP) by combining amplicon sequencing with metagenomics and mesocosm experiments. Detection of methyl-coenzyme M reductase subunit A (*mcrA*) gene amplicons demonstrated a wide diversity of Mcr-encoding archaea inhabit geothermal features with differing physicochemical regimes across YNP. From three selected hot springs we recovered twelve Mcr*-*encoding metagenome assembled genomes (MAGs) affiliated with lineages of cultured methanogens as well as *Candidatus* (*Ca*.) Methanomethylicia, *Ca*. Hadesarchaeia, and Archaeoglobi. These MAGs encoded the potential for hydrogenotrophic, aceticlastic, hydrogen-dependent methylotrophic methanogenesis, or anaerobic short-chain alkane oxidation. While Mcr-encoding archaea represent minor fractions of the microbial community of hot springs, mesocosm experiments with methanogenic precursors resulted in the stimulation of methanogenic activity and the enrichment of lineages affiliated with *Methanosaeta* and *Methanothermobacter* as well as with uncultured Mcr-encoding archaea including *Ca*. Korarchaeia, *Ca*. Nezhaarchaeia, and Archaeoglobi. We revealed that diverse Mcr-encoding archaea with the metabolic potential to produce methane from different precursors persist in the geothermal environments of YNP and can be enriched under methanogenic conditions. This study highlights the importance of combining environmental metagenomics with laboratory-based experiments to expand our understanding of uncultured Mcr-encoding archaea and their potential impact on microbial carbon transformations in geothermal environments and beyond.

## Introduction

**Fig. 1:**
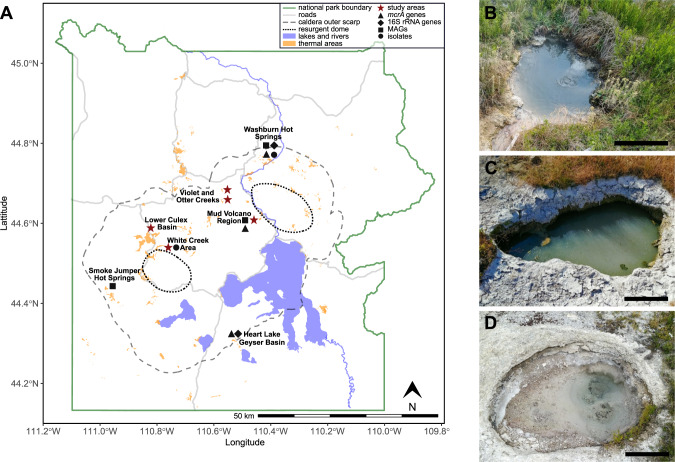
Geothermal areas of Yellowstone National Park and main study sites. **A** Map of Yellowstone National Park highlighting areas with potential for methanogenesis. Red stars mark areas investigated in this study. Symbols indicate where *mcrA* genes (triangle), 16S rRNA genes of methanogens (diamond), Mcr-encoding MAGs (square), or methanogen isolates (circle) have been recovered. Map was generated with data from the Wyoming State Geological Survey (https://www.wsgs.wyo.gov/pubs-maps/gis). Photographs of main study sites. LCB003, 2019-07-24 (**B**), LCB019, 2019-07-25 (**C**), and LCB024, 2019-07-23 (**D**). Scale bar indicates 30 cm.

Methane (CH_4_) is a climate active gas and an integral component in the global carbon cycle. The majority of biogenic methane is generated in anoxic environments by methanogenic archaea [1–3] that conserve energy by reducing low molecular weight substrates such as CO_2_, acetate, or methylated compounds to CH_4_ [4–8]. The final step in methanogenesis, the conversion of methyl-coenzyme M and coenzyme B into CH_4_, is catalyzed by the methyl-coenzyme M reductase (MCR) complex. This enzyme also catalyzes the reversible reaction, the activation of CH_4_ in anaerobic methane-oxidizing archaea [9] that, together with methanogens, control methane fluxes from anoxic environments, impacting global methane emissions to the atmosphere [1, 2]. Currently, all cultured methanogens belong to lineages within the Euryarchaeota, and their physiology and biochemistry has been studied for decades [10–15]. However, recent metagenomic studies discovered genes encoding the MCR complex in metagenome-assembled genomes (MAGs) from a variety of archaeal groups including *Candidatus* (*Ca*.) Methanofastidiosales, *Ca*. Nuwarchaeales, some members of the Archaeoglobi [16–18], as well as members of the TACK superphylum *Ca*. Methanomethylicia (*Ca*. Verstraetearchaeota), *Ca*. Korarchaeia, *Ca*. Bathyarchaeia, and *Ca*. Nezhaarchaeia [15, 19–22]. Some of these *mcrA* genes have been shown to be transcribed in situ but the function of the respective MCR complex has not been demonstrated [23–26]. Additionally, some MAGs contain highly divergent genes homologous to *mcr* [21, 22, 27–32]. These *mcr*-like genes encode an alkyl-coenzyme M reductase complex, which was experimentally shown to activate short-chain alkanes (i.e., ethane, propane, butane) in *Ca*. Synthrophoarchaeum, *Ca*. Argoarchaeum, and *Ca*. Ethanoperedens [27, 29, 31]. However, as most newly discovered Mcr-encoding archaea are yet uncultured, their proposed methanogenesis and anaerobic methane/alkane metabolism awaits further experimental evaluation. MAGs representing these archaea have frequently been recovered from anoxic and often high temperature geothermal environments, such as deep-sea hydrothermal vents and terrestrial hot springs [18–21, 27, 30, 33]. Geothermal environments have been used as model systems in microbial ecology for many decades. Their extreme nature and, as a consequence, reduced microbial complexity make them ideal for testing new technologies [34–36] and discovering new microbial lineages [37–39]. Methanogenesis in the geothermal system of Yellowstone National Park (YNP, Wyoming, USA), was initially studied in 1980 [40] and to date, three strains of the hydrogenotrophic methanogen *Methanothermobacter thermoautotrophicus* represent the only methanogens isolated from YNP [40, 41]. However, *mcrA* and 16S rRNA genes affiliated with methanogenic archaea and *mcr*-containing MAGs have been repeatedly recovered from geothermal features across YNP [20, 21, 24, 30, 33, 42], demonstrating the potential for a microbial methane cycle involving diverse archaeal lineages (Fig. 1A).

In this study, we further explored the potential for methanogenesis in YNP by [1] combining *mcrA* gene amplicon sequencing with aqueous geochemical measurements to identify methanogenic populations across 100 previously uncharacterized geothermal features, and [2] employing metagenomics and methanogenic mesocosm experiments to investigate the metabolic potential and activity of methanogenic populations in three selected hot springs. We describe the taxonomic diversity of *mcrA* genes detected across contrasting physicochemical conditions, detail the metabolic potential of *mcr*-containing MAGs, and reveal the responses of Mcr-encoding archaea to methanogenic precursor substrates.

## Materials and methods

### Selection of geothermal features and sample collection

A survey of *mcrA* gene diversity and aqueous geochemistry was conducted on 100 geothermal features including hot springs and mud pots with temperature between 18 and 94 °C and pH between 1.7 and 9.4 distributed across four geothermal regions in YNP (Fig. 1A). Because many of these geothermal features are not included in the YNP Research Coordination Network (http://rcn.montana.edu/), unique sample identifiers are used in this study, which indicate the area, feature, and DNA sample (e.g., LCB003.1 denotes DNA sample 1 from feature 003 in the Lower Culex Basin). Sediments and microbial mats for *mcrA* gene amplicon sequencing and shotgun metagenomics were collected in 2017 and 2018 using a stainless-steel cup, homogenized, and frozen immediately until DNA extraction. A slurry of sediment and water (1:9) for mesocosm experiments was obtained in 2019 (SI Tables 1–3) using a stainless-steel cup, transferred into a glass bottle, and homogenized. A 10 mL subsample was frozen immediately to preserve material for DNA extraction (environmental sample) before the bottle was sealed headspace-free. The slurry was transported in a heated container (~50 °C), placed at in situ temperature within 4 h and used to set up mesocosm experiments within 12 h of retrieval.

### Physicochemical measurements, aqueous geochemistry, and elemental analysis

Temperature and pH were recorded in the water column of geothermal features using a thermocouple and portable pH meter. Water samples for aqueous geochemistry were collected and analyzed for dissolved iron, sulfide, and gases (O_2_, CH_4_, CO_2_) as previously described [43–45]. Water samples for elemental analysis, anions, total carbon, inorganic carbon, non-purgeable organic carbon, and total nitrogen were processed by the Environmental Analytical Lab (Montana State University). Details in SI Materials and Methods.

### Mesocosm experiments

Mesocosm experiments with material from hot springs LCB003, LCB019, and LCB024 were prepared in an anoxic glove box (N_2_/CO_2_/H_2_; 90/5/5%). Under constant stirring, 10 mL aliquots of sediment slurry were distributed into 25 mL serum bottles using serological plastic pipettes. Mesocosms were set up with the following treatments: acetate, formate, H_2_, H_2_ plus CO_2_ and bicarbonate, methanol, monomethylamine, methanol plus H_2_, monomethylamine plus H_2_, paraformaldehyde (killed control), bromoethanesulfonate (methanogenesis inhibitor), and no amendment. Two sets of triplicates per treatment were performed: (1) with bacterial antibiotics streptomycin (inhibitor of protein synthesis) and vancomycin (inhibitor of peptidoglycan synthesis) and (2) without antibiotics. Liquid amendments were added to a final concentration of 5 mM except for paraformaldehyde (5% v/v) and antibiotics (50 mg/L). Serum bottles were sealed with butyl rubber stoppers and aluminum crimps before the headspace was exchanged with N_2_ (99.999%) at 100 kPa for 5 min and set to 200 kPa N_2_. H_2_ and CO_2_ were added by exchanging an equal volume of N_2_ for a final concentration of 50% H_2_ and/or 20% CO_2_. Mesocosms were incubated at in situ temperatures: 74 °C (LCB003), 55 °C (LCB019), 72 °C (LCB024). Mesocosm headspace gas composition was monitored by manually subsampling 2 mL at close to in situ temperature using a gastight syringe for analysis with a Varian gas chromatograph (GC; model CP2900) equipped with a dual-channel thermal conductivity detector system with Ar and He as carrier gases. Methane standards generated from 99.99% methane were measured at each timepoint. Triplicate mesocosms were terminated simultaneously when the first replicate reached a CH_4_ plateau or exhausted supplied H_2_. At this time, a 0.5 mL slurry subsample was pelleted (12,000 *g* for 5 min) and frozen for DNA extraction. Mesocosms with low or no methane production including controls were terminated after 43 (LCB003), 35 (LCB019), or 55 (LCB024) days.

### DNA extraction and gene amplification

DNA was extracted from environmental samples (1 mL) and mesocosm samples (pellet from 0.5 mL) using the FastDNA Spin Kit for Soil (MP Biomedicals, Irvine, CA) following the manufacturer’s guidelines. *mcrA* genes were amplified with primer set mlas-mod-F/mcrA-rev-R [46, 47] from environmental DNA extracts. Archaeal and bacterial 16S rRNA genes were amplified with the updated Earth Microbiome Project primer set 515F and 806R [48–50] from DNA extracts of the mesocosm experiment. Amplicon libraries were prepared as previously described [36] and sequenced by Laragen Inc. (Culver City, CA) or the Molecular Research Core Facility at Idaho State University (Pocatello, ID) using an Illumina MiSeq platform with 2 × 300 bp (*mcrA* amplicon library) and 2 × 250 bp (16S rRNA amplicon library) paired end read chemistry. Details in SI Materials and Methods.

### Amplicon sequence analysis

Both 16S rRNA and *mcrA* gene reads were processed using QIIME 2 version 2020.2 [51]. In short, primer sequences were removed from demultiplexed reads using cutadapt [52] with error rate 0.12, reads were truncated (145 bp forward, 145 bp reverse and 260 bp forward, 200 bp reverse for 16S rRNA and *mcrA* datasets, respectively), filtered, denoised, and merged in DADA2 with default settings [53]. 16S rRNA gene amplicon sequence variants (ASVs) were taxonomically classified with the sklearn method and the SILVA 132 database [54]. *mcrA* gene ASVs were assigned a taxonomy using vsearch with a minimum identity of 70% and no consensus classification against a reference database of representative near-full length *mcrA* genes encompassing the diversity of publicly available *mcrA*. Contamination was removed using the R package decontam [55]. The *mcrA* gene dataset was curated by removing ASVs ≤400 bp and non-*mcrA* gene ASVs as identified by evaluating the top hits of a blastx search against the NCBI NR database. Samples with less than 5000 reads or 10,000 reads for the 16S rRNA and *mcrA* gene dataset, respectively, were excluded from further analyses. Diversity metrics and Bray–Curtis dissimilarity were calculated with the R packages phyloseq [56] and vegan [57].

### Metagenome sequencing, assembly, and annotation

Metagenomes were generated at the Joint Genome Institute (JGI) from 10 ng (LCB003.1) and 100 ng (LCB019.1 and LCB024.1) DNA, and raw reads were processed according to JGI’s analysis workflow (see SI Materials and Methods). Quality controlled reads were assembled using SPAdes 3.11.1 [58] with options -m 2000, -k 33,55,77,99,127 -meta. Assembled scaffolds ≥2000 bp were binned with six implementations of four different programs including, Maxbin v2.2.4 [59], Concoct v1.0.0 [60], Metabat v2.12.1 [61], and Autometa v1 [62]. Bins generated from each program were refined with DAS_Tool [63] and bin quality statistics were determined with CheckM [64]. MAGs were assigned alphanumerical identifiers (e.g., LCB003-007 indicates bin 7 from feature 003 in the Lower Culex Basin) and MAGs containing at least one *mcrA* gene (>300 nt) and a complete or near-complete set of *mcrABGCD* were considered in this study. For these Mcr-encoding MAGs, annotations provided by the IMG/M-ER pipeline v7 [65] for genes associated with methanogenesis pathways, coenzyme and cofactor biosynthesis, energy conservation, and beta-oxidation, were manually evaluated using analysis of gene neighborhoods, NCBI BLASTP, NCBI’s Conserved Domain Database, TMHMM, InterPro, and the hydrogenase classifier HydDB [66–69]. See SI Data 3 for a complete list of gene annotations relevant to this study. Amino acid identity (AAI) values were computed with compareM using aai_wf and –proteins and taxonomies were assigned with GTDB-Tk v1.2.0 [70] and GTDB release 207 [71].

### Phylogenetic analyses

A set of 18 single-copy marker proteins [72, 73] detected in LCB Mcr-encoding MAGs and selected publicly available archaeal reference genomes (SI Table 4) were aligned using MUSCLE [74], trimmed with trimAL [75] with 50% gap threshold, and concatenated. A maximum likelihood phylogenetic tree was reconstructed with IQ-tree2 v2.0.6 [76] using the final concatenated alignment of 3916 positions, LG + F + R10 model, and 1000 ultrafast bootstraps.

McrA from LCB metagenomes (>100 aa), abundant ASVs (140 aa), and publicly available references were aligned with MAFFT-linsi [77], trimmed with trimAL with 50% gap threshold, and used for maximum likelihood phylogenetic analysis with IQtree2 with LG + C60 + F + G model and 1000 ultrafast bootstraps.

Sequence similarities between selected ASVs and 16S rRNA genes from metagenomes and MAGs were determined by blastn v2.13.0+.

## Results and discussion

### Survey of *mcrA* genes across physicochemically contrasting geothermal features

**Fig. 2:**
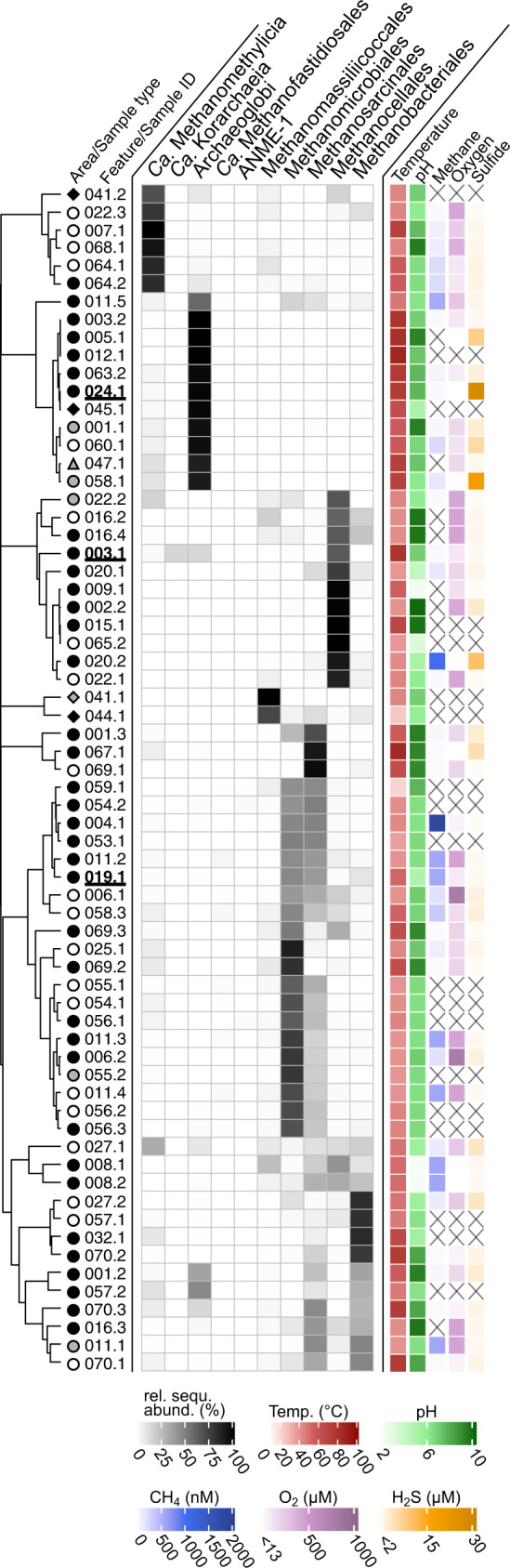
Diversity of mcrA genes detected in 66 samples from 39 geothermal features of YNP. Relative sequence abundance of *mcrA* gene amplicons affiliated with abundant lineages (relative sequence abundance >1% in at least one sample). Samples selected for metagenomics are underlined in bold. Samples were collected from geothermal features (identified by numbers) in the Lower Culex Basin (LCB, circle), Mud Volcano Region (MVR, diamond), and White Creek Area (WCA, triangle) and consisted of either sediment (black), microbial mat (white), or a mixture of sediment and mat material (grey). Physicochemical parameters of the geothermal water were recorded at the time of sample collection. X: no data available. Clustering based on Bray-Curtis dissimilarity using relative sequence abundance data of the presented lineages. No correlative trends between taxonomic affiliation of *mcrA* genes and physicochemistry were observed (SI Fig. 2). See SI Data 1 for details.

The presence and diversity of Mcr-encoding populations was assessed in 100 geothermal features of YNP via *mcrA* gene amplicon sequencing. Gene amplicons were recovered from 66 sediment and/or microbial mat samples spanning 39 geothermal features located in the Lower Culex Basin (LCB; 61 samples, 35 features), the Mud Volcano Region (MVR; 4 samples, 3 features), and the White Creek Area (WCA; 1 sample, 1 feature). These features were characterized by a wide range of temperature (22–86.3 °C), pH (2.40–9.77), and dissolved methane (40–1784 nM), oxygen (<13–771 µM), and sulfide (<2–27 µM; Figs. 1A, 2, SI Data 1).

Generally, the *mcrA*-containing microbial community in each geothermal feature was composed of a small number [1–21] of *mcrA* ASVs with >1% relative sequence abundance. The alpha diversity of *mcrA* tended to decrease with increasing temperature (SI Fig. 1, SI Data 1), a trend consistent with previous results based on 16S rRNA gene diversity in geothermal environments [42, 78, 79]. The *mcrA-*containing populations detected across samples included both confirmed methanogens (Methanomassiliicoccales, Methanosarcinales, Methanomicrobiales, Methanocellales, and Methanobacteriales) and lineages with proposed but untested methane/alkane metabolism (Archaeoglobi, *Ca*. Methanofastidiosales, *Ca*. Methanomethylicia, and *Ca*. Korarchaeia) (Fig. 2, SI Data 1). Confirmed methanogens dominated most samples (46/66) and were frequently identified in geothermal features with moderate temperatures (<60 °C). Other Mcr-encoding lineages prevailed at elevated temperatures (>60 °C) and were exclusively detected in LCB024.1, LCB058.1, and LCB063.2 (SI Fig. 2, SI Data 1). Particularly, Archaeoglobi-affiliated *mcrA* genes dominated at high temperatures (≥70 °C) and elevated concentrations of dissolved sulfide (≥3 µM; SI Fig. 2), which are conditions similar to those environments in which Archaeoglobi were previously detected [18, 21, 23, 80]. Notably, *Ca*. Korarchaeia were present at high relative sequence abundance (16%) in LCB003.1. Anaerobic methane-oxidizing archaea (ANME-1) as well as *Ca*. Methanofastidiosales were detected only with low relative sequence abundance (<2% and <4%, respectively; SI Data 1).

Overall, this survey of *mcrA* genes indicated that taxonomically diverse *mcrA-*containing archaea exist across a wide range of physicochemical regimes in the geothermal environments of YNP, particularly in the LCB geothermal area. As primer-based diversity surveys are inherently biased, we note that the primer set used in this study has historically been widely applied to amplify *mcrA* genes of Euryarchaeota origin [47]. While these primers bind to currently known *mcrA* genes of *Ca*. Methanomethylicia and *Ca*. Korarchaeia without mismatches, multiple mismatches to *mcrA* genes of other lineages exist (e.g., Ca. Nezhaarchaeia). Consequently, our amplicon-based gene survey likely underrepresented certain *mcrA* genes and underestimated *mcrA* gene diversity. To further investigate the methanogenic communities in the LCB, metagenomics and mesocosm experiments were conducted with material from hot springs LCB003, LCB019, and LCB024 characterized by elevated temperatures (47–73 °C) and circumneutral pH (3.0–7.8; SI Tables 1–3, Fig. 1B–D).

### *mcrA* gene diversity and *mcr*-containing MAGs recovered from the LCB

**Fig. 3:**
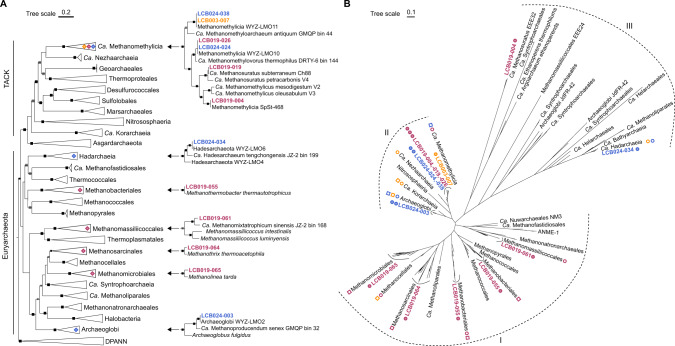
Phylogenetic tree of Mcr-encoding MAGs and McrA. **A** Maximum-likelihood tree, inferred with IQtree and the best-fit LG + F + R10 model, using a concatenated set of 18 conserved arCOGs (SI Table 4). Squares indicate ultrafast bootstrap values of 100 (black) and 95–99 (gray). Diamonds indicate lineages with Mcr-encoding MAGs detected in this study and shown in detail. **B** Maximum-likelihood tree, inferred with IQtree and the LG + C60 + F + G model, from the amino acid alignment of McrA. Filled circles: McrA identified in a MAG, open circles: metagenomic McrA (unbinned, >100 aa), open squares: abundant *mcrA* ASVs (>1% relative sequence abundance). For details see SI Table 6. Dashed line indicates previously proposed McrA/AcrA groups [21, 82]: I) McrA from methanogens and ANME (MCR-type), II) McrA from TACK lineages (MCR-type), III) McrA-like from proposed and experimentally confirmed alkane oxidizing archaea (ACR-type). Colors: orange, LCB003, magenta, LCB019, blue, LCB024.

In the hot springs selected as main study sites, abundant *mcrA* ASVs were related to confirmed methanogens in LCB019 and LCB003, and archaea with proposed methane/alkane metabolism in LCB024 and LCB003 (SI Table 6, SI Data 1). Environmental metagenomics recovered ten medium and two high quality MAGs [81] encoding McrA and a complete/near-complete MCR complex, which ranged in size from 0.73–1.78 Mbp and estimated completeness from 72 to 100% (Table 1). According to phylogenomic analysis of 18 archaeal single copy genes (SI Table 4), four MAGs belonged to lineages of previously cultured methanogens: *Methanothermobacter* (LCB019-055), *Methanomassiliicoccales* (LCB019-061), *Methanothrix* (LCB019-064), and *Methanolinea* (LCB019-065) while eight MAGs belonged to lineages of proposed methanogens or methane/alkane-oxidizing archaea: *Ca*. Methanomethylicia (LCB019-004, −019, −026, LCB024-024, −038, LCB003-007), Archaeoglobi (LCB024-003), and *Ca*. Hadesarchaeia (LCB024-034) (Fig. 3, SI Fig. 3). Interestingly, *Ca*. Methanomethylicia related MAGs were recovered from all three hot spring metagenomes indicating that members of this lineage can inhabit a wide range of physicochemical conditions. In contrast, MAGs affiliated with lineages of confirmed methanogens were only identified in LCB019, as initially reflected by *mcrA* amplicons. In total, 12 near-complete (≥500 aa) and 24 partial (100–499 aa) McrA sequences were recovered from the metagenome assemblies, suggesting that the Mcr-encoding MAGs reconstructed here do not reflect the full diversity and metabolic potential of the Mcr-encoding populations present. According to phylogenetic analysis, McrA proteins were categorized into MCR-type and ACR-type [21, 82] and affiliated with McrA of confirmed methanogens (group I), proposed methanogens (group II), or with McrA-like proteins of proposed alkane-metabolizing archaea (group III) [22, 27] (Fig. 3). Overall, the *mcrA* genes and Mcr-encoding MAGs recovered via metagenome sequencing confirmed the diversity of *mcrA-*containing archaea detected via amplicon sequencing and extended it by detecting Methanomassiliicoccales, *Ca*. Nezhaarchaeia, and *Ca*. Hadesarchaeia (Fig. 3).

**Table 1:** Statistics for twelve *mcr*-containing MAGs reconstructed from LCB metagenomes.

MAG	Taxonomy	Size (Mbp)	Contigs	Genes	Cov.	GC (%)	Compl. (%)	Redund. (%)	Abund. (%)	tRNAs	rRNAs 16S,23S,5S	*mcrA*	Quality
LCB019-065	*Methanolinea*	1.78	27	1847	587.6	57.9	98.7	0	2.33	17	1,1,1	1	Medium
LCB019-055	*Methanothermobacter*	1.55	217	1841	31.9	49.8	96.5	0.46	0.11	19	1,2,3	2	High
LCB019-064	*Methanothrix*	1.66	72	1674	491.4	54.6	100	0	1.82	16	0,0,2	1	Medium
LCB019-061	Methanomassiliicoccales	1.41	237	1501	24.3	44.3	87.61	3.23	0.08	13	1,0,1	1	Medium
LCB019-004	*Ca*. Methanomethylicia	1.17	216	1433	33.8	53.7	91	4.52	0.09	11	0,0,0	3	Medium
LCB019-019	*Ca*. Methanosuratus	1.09	182	1312	87.4	56.0	87.5	1.01	0.21	13	0,0,0	1	Medium
LCB019-026	*Ca*. Methanomethylovorales	1.48	111	1638	200.3	48.8	99.1	1.25	0.66	17	0,2,1	1^a^	Medium
LCB024-024	*Ca*. Methanomethylovorales	1.41	221	1673	64.9	49.6	88.5	2.1	0.24	16	0,0,1	1^a^	Medium
LCB003-007	*Ca*. Methanomethyloarchaeales	1.03	67	1243	26.7	27.9	93.5	0	0.11	15	0,0,1	1	Medium
LCB024-038	*Ca*. Methanomethyloarchaeales	1.17	53	1368	18.7	27.8	99.1	0.07	0.06	18	1,1,1	1	High
LCB024-003	Archaeoglobi	1.16	179	1399	87.2	45.7	88.5	1.31	0.27	18	0,1,0	2	Medium
LCB024-034	*Ca*. Hadesarchaeia	0.73	188	945	62.6	66	72.1	0.93	0.12	15	0,0,0	1	Medium

Taxonomy for MAGs was assigned using 18 marker proteins (SI Table 4). Average GC content, completeness, coverage, and redundancy were determined with CheckM. Total abundance was inferred based on the average coverage and size of each MAG relative to all MAGs in the metagenome.

^a^Indicates *mcrA* was split across two scaffolds. Quality was assessed according to ref. [81]. Cov., coverage; Compl., completeness; Redund., redundancy; Abund., total abundance.

### Potential for methane and alkane metabolism in *mcr*-containing MAGs

**Fig. 4:**
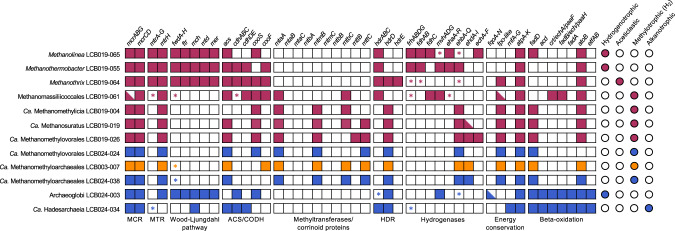
Methanogenic potential of the twelve Mcr-encoding MAGs. Squares indicate gene/gene set detected (filled), gene/gene set not detected (open) or gene set partially detected with the majority of genes present (half filled). * indicates only one gene in a gene set detected. Circles indicate the methane/alkane metabolism predicted for each MAG based on the gene repertoire. Colors: orange, LCB003, magenta, LCB019, blue, LCB024. A complete list of genes described in this figure and their abbreviations is reported in SI Data 3.

Four MAGs were affiliated with lineages of confirmed hydrogenotrophic, aceticlastic, and hydrogen-dependent methylotrophic methanogens and encoded *mcrA* genes related to those of cultured methanogens (group I). LCB019-065 and LCB019-055 shared amino acid identity (AAI) values of 80% and 98% with cultured representatives of the hydrogenotrophic methanogens *Methanolinea* and *Methanothermobacter*, respectively. Congruently, both MAGs encoded the genes required for generating methane from H_2_ and CO_2_, including the complete Wood-Ljungdahl Pathway (WLP), methyl-H_4_M(S)PT:coenzyme M methyltransferase (Mtr) complex, F_420_-reducing hydrogenase (Frh), methyl-viologen-reducing hydrogenase (Mvh) (incomplete in LCB019-065), and energy-converting hydrogenase (Ehb, LCB019-055; Ech, LCB019-065) (Fig. 4, SI Discussion). Additionally, a complete formate dehydrogenase complex (FdhABC) was encoded in LCB019-65 and while LCB019-055 encoded FdhAB, FdhC was not detected. Consistently, cultured representatives of *Methanolinea* utilize formate as a substrate for methanogenesis while those of *Methanothermobacter* do not [83, 84]. LCB019-064 showed AAI values of 90% to the aceticlastic methanogen *Methanothrix thermoacetophila* and encoded all genes necessary for aceticlastic methanogenesis including the Mtr complex and acetyl-CoA decarbonylase/synthase:CO dehydrogenase complex (ACS/CODH) (Fig. 4, SI Discussion). LCB019-061 shared AAI of 59% with cultured *Methanomassiliicoccus* sp., suggesting it may represent a novel lineage within the Methanomassiliicoccales. Consistent with a hydrogen-dependent methylotrophic methanogenesis lifestyle of Methanomassiliicoccales isolates, LCB019-061 encodes methyltransferases (SI Fig. 4) but lacks the WLP and a complete Mtr complex. A *mtrH* gene encoded in proximity to methyltransferase corrinoid activation protein (*ramA*) suggests LCB019-061 may reduce unknown methylated substrates to methane [19, 80].

Six MAGs shared high AAI values (>96%) with MAGs of *Ca*. Methanomethylicia and encoded an McrA affiliated with those of other *Ca*. Methanomethylicia MAGs (group II). Consistent with *Ca*. Methanomethylicia MAGs proposed to perform hydrogen-dependent methylotrophic methanogenesis [19], the six MAGs lack the WLP and a complete Mtr complex but encode a variety of methyltransferases including methanol:coenzyme M methyltransferase (*mtaA)*, monomethylamine methyltransferase (*mtmB*), and dimethylamine corrinoid (*mtbC*) and/or trimethylamine corrinoid protein (*mttC*). LCB019-026 additionally encoded a trimethylamine methyltransferase (*mttB*). A methyltransferase subunit H of the Mtr complex, *mtrH*, encoded near other corrinoid protein and methyltransferase genes (SI Data 3) suggests that methane may be formed from unknown methylated substrates [19, 80]. Although methylamine-specific cobamide:coenzyme M methyltransferase (*mtbA*) was not identified, MtaA could substitute for the activity of MtbA (SI Fig. 4) [85]. Thus, all six *Ca*. Methanomethylicia MAGs contain the gene repertoire needed for hydrogen-dependent methylotrophic methanogenesis (Fig. 4, SI Fig. 4, SI Discussion). In addition, LCB019-004 encoded a second McrA, that clustered with the McrA-like proteins of ethane-oxidizing archaea *Ca*. Ethanoperedens and *Ca*. Argoarchaeum (McrA group III, ACR/ECR type) and a recently recovered MAG of *Ca*. Methanosuratus [25, 82] proposed to perform ethanogenesis or ethane oxidation via an unknown pathway [82]. This indicates that anaerobic methane/alkane metabolism within the *Ca*. Methanomethylicia may be more diverse than previously anticipated.

The Archaeoglobi affiliated MAG LCB024-003 showed low AAI values (65%) to *Archaeoglobales* isolates, which are all non-methanogenic sulfate-reducers. Instead, LCB024-003 shared high AAI values (>98%) to Mcr-encoding Archaeoglobi MAGs of proposed hydrogenotrophic methanogens (WYZ-LMO10, SJ34) or hydrogen-dependent methylotrophic methanogens (*Ca*. M. hydrogenotrophicum) [21, 33, 80]. Consistently, its two partial McrA (192 and 193 aa) cluster with McrA of other proposed methanogenic Archaeoglobi (group II) (Fig. 3) [18, 21]. LCB024-003 encodes genes required for hydrogenotrophic methanogenesis including the WLP pathway, hydrogenase Mvh, and a F_420_H_2_:quinone oxidoreductase complex (*fqo*DHIF) which may substitute for Frh to generate reduced F_420_ as previously suggested [23, 86, 87]; however, a complete Mtr complex was not detected. In contrast to *Ca*. M. hydrogenotrophicum, LCB024-003 encodes a truncated 5,10-methylenetetrahydromethanopterin reductase (*mer*) while *mtaABC* were not identified, suggesting it is unable to use methanol for methanogenesis [23]. Although LCB024-003 encodes the beta-oxidation pathway, other genes typically associated with short-chain alkane oxidation including an ACR-type MCR, ACS/CODH complex, and methyltransferases were absent. Hence, unlike the MAGs of *Ca*. Polytropus marinifundus and JdFR-42 [18, 21], LCB024-003 may not represent an anaerobic alkane oxidizer [88] and instead may utilize the beta-oxidation pathway for long chain fatty acid metabolism as has been shown for *Archaeoglobus fulgidus* [89]. Further, genes encoding dissimilatory sulfate reduction (*sat, aprAB, dsrABC*) present in some *mcr-*containing Archaeoglobi MAGs (*Ca*. M. dualitatem [23]) were not detected. Together, the genomic information from LCB024-003 suggests that this Archaeoglobi representative may live as a hydrogenotrophic methanogen (Fig. 4, SI Discussion).

LCB024-034 shared AAI values of >79% with other Mcr-encoding Hadesarchaeia [21, 80] and encoded a partial McrA (216 aa) related to the ACR-type proteins of Hadesarchaeia (group III) [27]. Congruently with the hypothesis of short-chain alkane metabolism in *Ca*. Hadesarchaeia, LCB024-034 encoded the beta-oxidation pathway and an ACS/CODH complex. However, most genes encoding the WLP required for oxidizing activated alkanes to CO_2_ were missing [27]. Thus, short-chain alkane metabolism in LCB024-034 remains speculative, awaiting further genomic and experimental data.

Together, the 12 *mcr-*containing MAGs reconstructed here reflect the potential for archaeal short-chain alkane-oxidation as well as hydrogenotrophic, aceticlastic, and hydrogen-dependent methylotrophic methanogenesis in geothermal environments of YNP. Further, these MAGs extend the genomic data available for future analysis of diversity and evolution of Mcr-encoding archaea and suggest geothermal environments are a promising source for the recovery of these archaea.

### Methanogenic activity and enrichment of methanogens in mesocosms

**Fig. 5:**
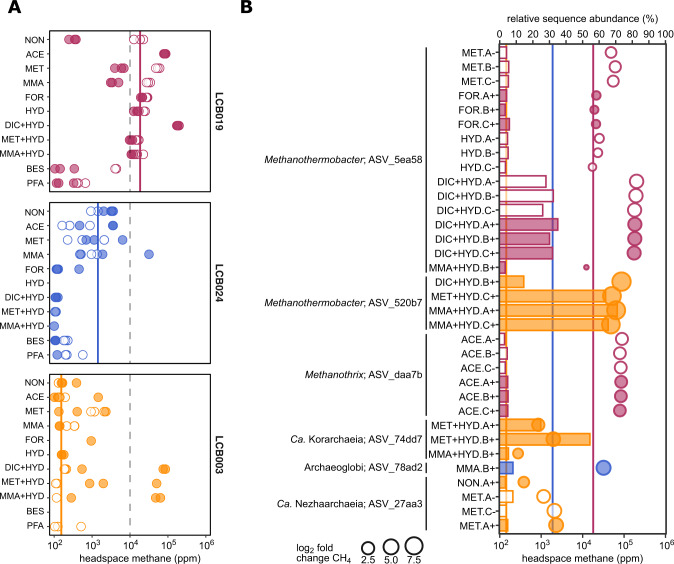
Methane production and enrichment of Mcr-encoding archaea in mesocosms. **A** Maximum methane produced in the headspace of mesocosms. Replicates measuring <100 ppm not shown. **B** Enrichment of 16S rRNA gene ASVs (>3% relative sequence abundance) affiliated with Mcr-encoding archaea across treatments (bars) paired with respective headspace methane yields (circles). Circle size proportional to the log2 fold change in methane yield between treatment and control (i.e., mesocosm under close to in situ condition) for each site. Dashed lines indicate 1% methane. Solid lines indicate average methane concentration in mesocosms under close to in situ conditions (no substrate, no antibiotics) for each site. Open symbols: without antibiotics; filled symbols: with antibiotics. Colors: orange, LCB003, magenta, LCB019, blue, LCB024. Abbreviations: NON, no amendment control; ACE, acetate; MET, methanol; MMA, monomethylamine; FOR, formate; HYD, hydrogen (H_2_); DIC, dissolved inorganic carbon (HCO_3_^−^ + CO_2_); BES, bromoethanesulfonate (methanogenesis inhibitor); PFA, paraformaldehyde (killed control). Replicates indicated as A-C and+, with antibiotics or -, without antibiotics. Methane curves and extended relative abundance data for all mesocosm replicates are reported in SI Figs. 5–7 and SI Data 4.

Mesocosm experiments were performed to reveal activity and enrichment of methanogens. Methane accumulation was monitored in the headspace of mesocosms under (1) close to in situ conditions (i.e., no amendment), (2) conditions favoring methanogenesis (i.e., substrate amendment), and (3) conditions inhibiting bacterial metabolism (i.e., antibiotics treatment) (SI Fig. 5). Inhibition of bacterial metabolism may have disrupted potential symbiotic partnerships between methanogens and bacteria and/or favored substrate availability for methanogens through the limitation of competition. Mesocosms were also analyzed for enrichment in potential methanogenic populations via 16S rRNA gene amplicon sequencing. Abundant 16S rRNA gene ASVs (>1% relative sequence abundance) related to Mcr-encoding archaea amounted for ~1% in LCB019 and <1% in LCB024 and LCB003, indicating that methanogens represent a minor fraction of the in situ community (SI Figs. 6, 7). However, in mesocosms from all three hot springs, methane production was observed under close to in situ conditions with strongly varying maximum methane yields (17,000, 1900, and 150 ppm for LCB019, LCB024, and LCB003, respectively; Fig. 5, SI Fig. 5). Substrate amendment had considerably different effects on methane production and, except for LCB019, mesocosm triplicates showed strong variation and long response times (20–40 days) likely due to an uneven distribution of initially low abundant methanogen cells across replicates. For LCB024, substrate amendment (particularly H_2_), appeared to suppress methane production, which may indicate that either hydrogenotrophic methanogens were not present, not active, or were outcompeted by other community members considering the shift in the microbial community (SI Fig. 7). Antibiotic amendments resulted, on average across treatments, in increased methane production in mesocosms from LCB024 and LCB003, and a strong decrease in methane production in mesocosms from LCB019, indicative of substrate competition or metabolic interdependencies between methanogens and bacteria, respectively.

To characterize the effect of substrate amendment on methanogenic populations, we analyzed ASVs related to Mcr-encoding archaea with enrichment >3% relative sequence abundance across treatments. H_2_ plus DIC (HCO_3_^−^ + CO_2_) amended mesocosms from LCB019 showed rapid methane production, with highest maximum methane concentrations (>170,000 ppm) reached within 6 days (SI Fig. 5). These mesocosms were enriched (26–35%) in ASV_5ea58, identical to the 16S rRNA gene of MAG LCB019-055 as well as *Methanothermobacter thermautotrophicus*, a thermophilic hydrogenotrophic methanogen isolated from YNP [40, 41]. Similarly, for LCB003, H_2_ plus DIC or methylated compounds resulted in the strongest stimulation of methanogenesis and most pronounced enrichment (up to 71%) of an ASV affiliated with *Methanothermobacter crinale* (ASV_520b7, 99.6% sequence identity). Notably, H_2_ amendment without DIC supply did not result in a comparable response, suggesting that in closed mesocosm systems hydrogenotrophic methanogens were limited by inorganic carbon, which unlikely occurs in situ where concentrations of aqueous CO_2_ were elevated (SI Table 2). For LCB019, acetate amendment resulted in elevated methane production and concomitant enrichment (3–5%) of ASV_daa7b, which shared high sequence similarity with the aceticlastic methanogen *Methanothrix thermoacetophila* (98%) and a 16S rRNA gene recovered from the LCB019 metagenome (100%; SI Data 4). MAG LCB019-064, related to *Methanothrix thermoacetophila* (89% AAI similarity) encoded the potential for methanogenesis from acetate and may represent the enriched *Methanothrix* sp. population. Thus, our mesocosm experiments complemented findings from metagenomics, confirming the potential for hydrogenotrophic methanogenesis by *Methanothermobacter* sp. and aceticlastic methanogenesis by *Methanothrix* sp. in LCB019 and revealing the potential for hydrogenotrophic methanogenesis by *Methanothermobacter* sp. in LCB003 (SI Table 6).

In addition to previously cultured methanogens, uncultured Mcr-encoding lineages were enriched. An ASV identified as *Ca*. Methanodesulfokores washburnensis (ASV_74dd7, 100% sequence identity) was highly abundant (25–54%) in two mesocosms from LCB003 amended with methanol, hydrogen, and antibiotics. A MAG of this *Ca*. Korarchaeia representative previously recovered from YNP encodes versatile metabolic capabilities including hydrogen-dependent methylotrophic methanogenesis from methanol [20]. Methane yields in these mesocosms, while comparably low after 43 days (<2000 ppm), were strongly elevated compared to the no amendment control of LCB003 (log2 fold change (FC) 3–4). *mcrA* and 16S rRNA genes of *Ca*. Methanodesulfokores washburnensis were also detected via amplicon and metagenome sequencing, confirming the presence of this lineage in LCB003 (Figs. 2, 3, SI Data 4). In one mesocosm from LCB024 amended with monomethylamine, stimulation of methanogenesis (log2 FC 4, 310,000 ppm) and enrichment (8%) of Archaeoglobi-affiliated ASV_78ad2 was observed. The Archaeoglobi MAG LCB024-003 recovered from LCB024 encoded the potential for hydrogenotrophic methanogenesis while genes required for methylotrophic methanogenesis were not detected (Fig. 4). However, potential for methylotrophic methanogenesis has been described for some Archaeoglobi MAGs and the recovery of several Archaeoglobi related *mcrA* and 16S rRNA genes from LCB024 suggests that diverse Archaeoglobi populations are present, possibly including methylotrophic methanogens. An enrichment of a *Ca*. Nezhaarchaeia related ASV (ASV_27aa3) was highest (8%) in methanol amended mesocosms from LCB003 and cooccurred with elevated methane yields (log2 FC 3.5, 85,000 ppm), confirming the persistence of a *Ca*. Nezhaarchaeia population detected by metagenomic 16S rRNA and *mcrA* genes (SI Data 2). Previously described *mcr*-containing *Ca*. Nezhaarchaeia MAGs encode the potential for hydrogenotrophic methanogenesis, and while no enrichment was detected in hydrogen amended mesocosms, microbially produced hydrogen may have facilitated limited methanogenic activity and enrichment of hydrogenotrophic methanogens in other mesocosms. *Ca*. Methanomethylicia related ASVs were detected in multiple mesocosms, however their enrichment remained low (<3%) (SI Data 4).

Overall, minor methanogenic populations, not or hardly detectable in hot springs via 16S rRNA gene or metagenome sequencing, were enriched in mesocosm experiments under selective methanogenic conditions. Specifically, acetate or hydrogen plus DIC enabled the enrichment of *Methanothrix* or *Methanothermobacter*, respectively, while methyl compounds favored the enrichment of *Ca*. Korarchaeia, *Ca*. Nezhaarchaeia, or Archaeoglobi. Further research is needed to decipher the metabolism of the here enriched populations of uncultured archaea, their proposed methanogenic capacities, and potential metabolic interdependencies with other community members.

### Implications for methane cycling in YNP

We explored the potential for methanogenesis in previously uncharacterized geothermal environments of YNP, primarily the LCB, and our results warrant further research into the magnitude of biological methane production in this area. While the methanogenic communities of eight geothermal features in YNP had previously been investigated [20, 21, 24, 30, 33, 42] we detected *mcrA* genes across an additional 39 geothermal features indicating the wide distribution of diverse populations of Mcr-encoding archaea, including both confirmed methanogens and lineages proposed to engage in anaerobic methane/alkane cycling. The methanogenic pathways encoded across *mcr*-containing MAGs suggests methanogenesis in LCB hot springs could proceed from different precursors including H_2_/CO_2_, acetate, and methyl compounds plus hydrogen. The genetic potential for hydrogen-dependent methylotrophic methanogenesis was encoded by the majority of MAGs, including *Ca*. Methanomethylicia and Methanomassiliicoccales, and was detected in all three hot springs, possibly reflecting prevalence of this metabolism in geothermal environments as previously proposed [80]. While methanogenic populations accounted for minor fractions of the microbial community, methanogenesis may proceed in situ as it was observed in mesocosms under close to in situ conditions. The potential for hydrogenotrophic and aceticlastic methanogenesis revealed by metagenomics was confirmed by the enrichment of *Methanothermobacter* and *Methanothrix* in mesocosms under selective substrate amendment. In situ, methanogenesis in hot springs is likely constrained by physicochemical regimes, substrate availability, and metabolic interdependencies. Methanogenic precursors may be supplied from organic matter degradation as metabolic intermediates of syntrophic communities (e.g., H_2_, acetate), products of respiration (e.g., CO_2_), or through geothermal alteration from the subsurface (e.g., H_2_, CO_2_) [40, 90]. As hot springs often present dynamic systems, methanogens may frequently respond with activity and growth to favorable conditions. This may be exemplified by *Methanothermobacter*’s capacity to rapidly respond, resulting in high activity and fast growth upon supply of H_2_/CO_2_, which it may sporadically or consistently encounter in situ (Fig. 5, SI Table 2, SI Fig. 5).

Although methanogenic activity and isolation of *Methanothermobacter thermoautotrophicus* have been demonstrated [40, 41], the environmental impact of methanogens on methane emissions from YNP’s geothermal environments is not well understood. Methane is an important component of the gas flux in YNP [90–92] and the isotopic composition of gas emitted from geothermal features across YNP has suggested methane is primarily generated through abiogenic and/or thermogenic processes, while methanogenesis is not a significant source of methane [91]. Although we detected varying concentrations of aqueous methane in geothermal features in an area of YNP that had not been previously investigated, the source and fate of this methane is currently unknown. In general, methane emissions from terrestrial geothermal environments are not considered in estimates of the global atmospheric methane budget and little is known about their contribution to the global methane flux [1, 3, 14]. YNP contains more than 14,000 geothermal features, the largest concentration in the world, making it a superior candidate for studying CH_4_ flux in these environments [93–95].

Environmental *mcrA* gene surveys and metagenomics aid in identifying environments in which methanogenesis may occur. Subsequent quantification of in situ metabolic activities, including methane production rates, as well as deciphering the interplay between methanogens and methanotrophs will lead to a better understanding of the impact methanogens have on the local carbon cycle and their contribution to methane emissions from YNP’s geothermal environments.

## Conclusion

Uncultured Mcr-encoding lineages are globally distributed across a wide range of ecosystems and could play important roles in the biogeochemical carbon cycle [14, 18–24, 28, 30, 33]. In this study, we described a previously unrecognized diversity of Mcr-encoding archaea in geothermal environments of YNP. Environmental metagenomics provided insights into the metabolic potential of these Mcr-encoding archaea and mesocosm experiments revealed, for some lineages for the first time, their activity and enrichment under methanogenic conditions. The ability to enrich these uncultured Mcr-encoding archaea in laboratory settings presents a clear path towards their cultivation. Future work, including experiments under close to in situ conditions and culture-dependent physiology and biochemistry studies, will be essential for advancing our understanding of these still widely enigmatic archaea.

## Supplementary Information

Supplementary Information

SI Data 1

SI Data 2

SI Data 3

SI Data 4

## Data Availability

16S rRNA gene and *mcrA* gene amplicon data as well as *mcr-*containing MAGs are deposited at NCBI under BioProject PRJNA859922 (SI Table 5). Metagenomes are available on IMG/M (JGI) under IMG Genome IDs 3300028675 (LCB003), 3300031463 (LCB019), and 3300029977 (LCB024).
