# Supplementary material for: Diversity and function of methyl-coenzyme M reductase-encoding archaea in Yellowstone hot springs revealed by metagenomics and mesocosm experiments
Source: ISME Commun. 2023 Mar 22;3:22. doi: 10.1038/s43705-023-00225-9 (PMC10033731; doi:10.1038/s43705-023-00225-9)
Supplement: Supplementary file 1 — Supplementary Information [file 43705_2023_225_MOESM1_ESM.pdf]

**Supplementary Information**

# Diversity and function of Methyl-coenzyme M reductase-encoding archaea in Yellowstone hot springs revealed by metagenomics and mesocosm experiments

Mackenzie M. Lynes[*], Viola Krukenberg[*,#], Zackary J. Jay, Anthony J. Kohtz, Christine A. Gobrogge, Rachel L. Spietz, Roland Hatzenpichler[#]

*These authors contributed equally to this work.

#Correspondence to viola.krukenberg@montana.edu and roland.hatzenpichler@montana.edu.

**Table of contents**

**SI Table 1.** Location, pH, and temperature of main study sites.
**SI Table 2.** Aquatic geochemistry of main study sites (2017-2020).
**SI Table 3.** Elemental analysis of main study sites (2017).
**SI Table 4.** Marker genes used in phylogenomic analysis of MAGs.
**SI Table 5.** NCBI BioSamples for MAGs under BioProject PRJNA859922.
**SI Table 6.** Overview of Mcr-encoding lineages detected in this study.
**SI Figure 1.** Alpha diversity of *mcrA* gene ASVs across temperature and pH.
**SI Figure 2**. Distribution of Mcr-encoding archaea across physicochemical regimes.
**SI Figure 3.** Phylogenetic affiliation of Mcr-encoding MAGs.
**SI Figure 4.** Proposed pathways for methylotrophic methanogenesis in MAGs from this study.
**SI Figure 5.** Development of methane concentrations in the headspace of mesocosms.
**SI Figure 6.** Relative sequence abundance of archaeal ASVs detected at >1% in mesocosms.
**SI Figure 7.** Relative sequence abundance of phyla detected at >3% in mesocosms.
**Extended Materials and Methods.**
**Extended Discussion.**
**References.**
**SI Data 1.** Environmental survey of physicochemistry and *mcrA* genes. See separate excel file.
**SI Data 2.** Additional information for Mcr-encoding MAGs. See separate excel file.
**SI Data 3.** Gene inventory for Mcr-encoding MAGs. See separate excel file.
**SI Data 4.** Mesocosm 16S rRNA gene amplicon data. See separate excel file.

**SI Table 1.** Location, pH, and temperature of main study sites. Data recorded at the time of sampling for metagenomics (2017) and mesocosm experiments (2019).

| | Year | Unit | LCB003 | LCB019 | LCB024 |
|---|---|---|---|---|---|
| Latitude | | N. Lat | 44.57763 | 44.56765 | 44.57347 |
| Longitude | | W. Lon | 110.78957 | 110.80759 | 110.79504 |
| pH | 2017 | | 6.47 | 3.02 | 7.79 |
| Temperature | | ˚C | 72.5 | 47.1 | 69.4 |
| pH | 2019 | | 6.55 | 5.25 | 7.00 |
| Temperature | | ˚C | 72.5 | 47.0 | 69.3 |

**SI Table 2.** Aquatic geochemistry of main study sites (2017-2020). Data shown as ranges when applicable.

| | Year | Unit | LCB003 | LCB019 | LCB024 |
|---|---|---|---|---|---|
| pH | 2017-2020 | | 6.14 - 6.84 | 3.02 - 5.51 | 7.00 - 7.90 |
| Temperature | | ˚C | 70.1 - 79.0 | 44.4 - 52.3 | 61.4 - 73.7 |
| $CH_4$ (aq) | 2019-2020 | nM | 40 - 211 | 470 - 576 | 30 - 77 |
| $O_2$ (aq) | | µM | <13 - 117 | <13 - 171 | <13 - 131 |
| $H_2S$ (aq) | | µM | 0.91 - 3.28 | 1.04 - 14.59 | 11.98 - 34.50 |
| Fe (II) | | µM | 0 - 12 | 82 - 104 | 0 |
| total Fe (II, III) | | µM | 0 - 20 | 97 - 99 | 0 - 1.1 |
| $CO_2$ (aq) | | µM | 27 - 340 | 487 - 580 | 16 - 32 |
| $PO_4^{3-}$ | 2017 | µM | 0.98 | 6.11 | 0.79 |
| total N | | µM | 19.70 | 56.78 | 9.55 |
| $NH_4^+$ | | µM | 6.26 | 29.14 | 1.54 |
| $NO_2^-$ | | µM | 0.05 | 0.04 | 0.24 |
| $SO_4^{2-}$ | | mM | 0.14 | 0.36 | 0.15 |
| $Cl^-$ | | mM | 8.03 | 4.88 | 8.53 |
| $F^-$ | | mM | 1.28 | 0.95 | 1.53 |
| total C | | mM | 2.57 | 2.56 | 2.96 |
| inorganic C | | mM | 1.87 | 0.89 | 2.97 |
| non-purgeable organic C | | mM | 0.18 | 0.30 | 0.07 |

**SI Table 3.** Elemental analysis of main study sites (2017). BD, below detection.

| | Unit | LCB003 | LCB019 | LCB024 |
|---|---|---|---|---|
| Ag | µM | 0.01 | BD | 0.06 |
| Al | µM | 8.34 | 44.48 | 9.45 |
| As | µM | 11.41 | 5.81 | 15.42 |
| Ba | µM | BD | 0.26 | BD |
| Be | µM | 1.00 | BD | BD |
| Bi | µM | 0.07 | BD | BD |
| Ca | µM | 52.40 | 67.37 | 28.07 |
| Cd | µM | BD | BD | BD |
| Co | µM | BD | BD | BD |
| Cr | µM | BD | BD | BD |
| Cu | µM | BD | BD | BD |
| Fe | µM | 1.37 | 6.98 | BD |
| Mg | µM | 11.73 | 22.83 | BD |
| Mn | µM | BD | 0.38 | BD |
| Mo | µM | 0.27 | BD | 1.72 |
| Ni | µM | BD | BD | BD |
| Pb | µM | 0.06 | BD | BD |
| Sb | µM | BD | BD | 0.17 |
| Se | µM | 0.28 | 0.09 | 0.11 |
| Sr | µM | BD | BD | BD |
| Ti | µM | 0.47 | BD | BD |
| Tl | µM | 0.01 | BD | BD |
| V | µM | 0.53 | 0.06 | 0.27 |
| Zn | µM | 0.53 | 0.16 | 0.11 |
| B | mM | 0.33 | 0.17 | 0.34 |
| K | mM | 0.13 | 0.35 | 0.25 |
| Li | mM | 0.33 | 0.23 | 0.40 |
| Na | mM | 10.25 | 5.09 | 12.16 |
| S | mM | 0.31 | 0.73 | 0.26 |
| Si | mM | 2.47 | 3.07 | 3.09 |

**SI Table 4.** NCBI BioSamples for MAGs under BioProject PRJNA859922.

| **MAG** | **Taxonomy** | **NCBI Taxonomy/Organism** | **BioSample** | **Accession** |
|---|---|---|---|---|
| LCB019-065 | *Methanolinea* | *Methanolinea* sp. | SAMN29855412 | JANIHI000000000 |
| LCB019-055 | *Methanothermobacter* | *Methanothermobacter* sp. | SAMN29855422 | JANIHH000000000 |
| LCB019-064 | *Methanothrix* | *Methanothrix* sp. | SAMN29855467 | JANIHG000000000 |
| LCB019-061 | Methanomassiliicoccales | Methanomassiliicoccales archaeon | SAMN29855483 | JANHAK000000000 |
| LCB019-004 | *Ca.* Methanomethylicia | *Ca*. Methanomethylicia archaeon | SAMN29877625 | JANHAI000000000 |
| LCB019-019 | *Ca.* Methanosuratus | *Ca*. Methanosuratincola sp. | SAMN29877776 | JANIHJ000000000 |
| LCB019-026 | *Ca.* Methanomethylovorales | *Ca*. Methanomethylicia archaeon | SAMN29877790 | JANHAJ000000000 |
| LCB024-024 | *Ca.* Methanomethylovorales | *Ca*. Methanomethylicia archaeon | SAMN29851525 | JANHAL000000000 |
| LCB003-007 | *Ca.* Methanomethyloarchaeales | *Ca*. Methanomethylicia archaeon | SAMN29878106 | JANHAH000000000 |
| LCB024-038 | *Ca.* Methanomethyloarchaeales | *Ca*. Methanomethylicia archaeon | SAMN29851763 | JANHAM000000000 |
| LCB024-003 | Archaeoglobi | Archaeoglobi archaeon | SAMN29809817 | JANFND000000000 |
| LCB024-034 | *Ca.* Hadesarchaeia | *Ca.* Hadesarchaea archaeon | SAMN29854158 | JANIHF000000000 |

**SI Table 5.** Marker genes used in phylogenomic analysis of MAGs.

| arCOG | Gene | Description |
|---|---|---|
| arCOG00415 | RecA | RecA/RadA recombinase |
| arCOG01227 | FtsY | Signal recognition particle GTPase |
| arCOG01762 | RpoB/Rpo2 | DNA directed RNA polymerase subunit B |
| arCOG01946 | RPS6A | Ribosomal protein S6E (S10) |
| arCOG01950 | RPL24A | Ribosomal protein L24E |
| arCOG04067 | RplB | Ribosomal protein L2 |
| arCOG04072 | RplW | Ribosomal protein L23 |
| arCOG04087 | RpsE | Ribosomal protein S5 |
| arCOG04088 | RplR | Ribosomal protein L18 |
| arCOG04097 | RpsC/rps3p | Ribosomal protein S3 |
| arCOG04098 | RplV/rpl22p | Ribosomal protein L22 |
| arCOG04099 | RpsS/rps19p | Ribosomal protein S19 |
| arCOG04185 | RpsO | Ribosomal protein S15P |
| arCOG04208 | RPL43A | Ribosomal protein L37AE/L43A |
| arCOG04255 | RpsL/rps12p | Ribosomal protein S12 |
| arCOG04277 | Efp | Translation elongation factor P (EF-P)/translation initiation factor 5A (eIF-5A) |
| arCOG04314 | RPS28A | Ribosomal protein S28E/S33 |
| arCOG04372 | RplK | Ribosomal protein L11 |

**SI Table 6.** Overview of Mcr-encoding lineages detected in this study. Color coding by site: orange, LCB003, magenta, LCB019, blue, LCB024.

| **Detected group** | **Status** | **MCR-based metabolism/ metabolic potential** | *mcrA* ASVs[1] | | | *mcrA* MG[2] | | | *mcrA* MAG[3] | | | 16S rRNA ASVs[4] | | |
|---|---|---|---|---|---|---|---|---|---|---|---|---|---|---|
| | | | LCB003 (orange) | LCB019 (magenta) | LCB024 (blue) | LCB003 (orange) | LCB019 (magenta) | LCB024 (blue) | LCB003 (orange) | LCB019 (magenta) | LCB024 (blue) | LCB003 (orange) | LCB019 (magenta) | LCB024 (blue) |
| *Methanocella* | cultured | methanogenesis ($H_2$) | 2 | | | | 3 | | | | | | | |
| *Methanothrix* | cultured | methanogenesis (acetate) | | 2 | | | 1 | | | 1 | | | 1 | |
| *Methanolinea* | cultured | methanogenesis ($H_2$) | | 2 | | | 1 | | | 1 | | | | |
| *Methanothermobacter* | cultured | methanogenesis (methyl- groups) | | 3 | | | 5 | | | 1 | | 1 | 2 | |
| Methanomassiliicoccales | cultured | methanogenesis ($H_2$, methyl-groups) | | | | | 2 | | | 1 | | | | |
| Archaeoglobi | MAG | methanogenesis ($H_2$, methyl-groups), methane/alkane oxidation | 3 | | 5 | | | 5 | | | 1 | 3 | | 1 |
| *Ca.* Korarchaeia | MAG | methanogenesis ($H_2$, methyl-groups), methane oxidation | 1 | | | 1 | | | | | | 1 | | |
| *Ca.* Methanomethylicia | MAG | methanogenesis ($H_2$, methyl-groups), methane/alkane oxidation | | | 1 | 2 | 6 | 2 | 1 | 4 | 1 | | 2 | |
| *Ca.* Nezhaarchaeia | MAG | methanogenesis ($H_2$) | | | | 2 | | | | | | 1 | | |
| *Ca.* Hadesarchaeia | MAG | methane/alkane oxidation | | | | 1 | 2 | 3 | | | 1 | | | |

[1] number of *mcrA* ASVs at >1% relative sequence abundance detected via amplicon sequencing.
[2] number of *mcrA* genes (>300 nt) detected in metagenomes.
[3] number of reconstructed MAGs containing *mcrA* genes.
[4] number of 16S rRNA ASVs enriched in mesocosms at >3% relative sequence abundance detected via amplicon sequencing.

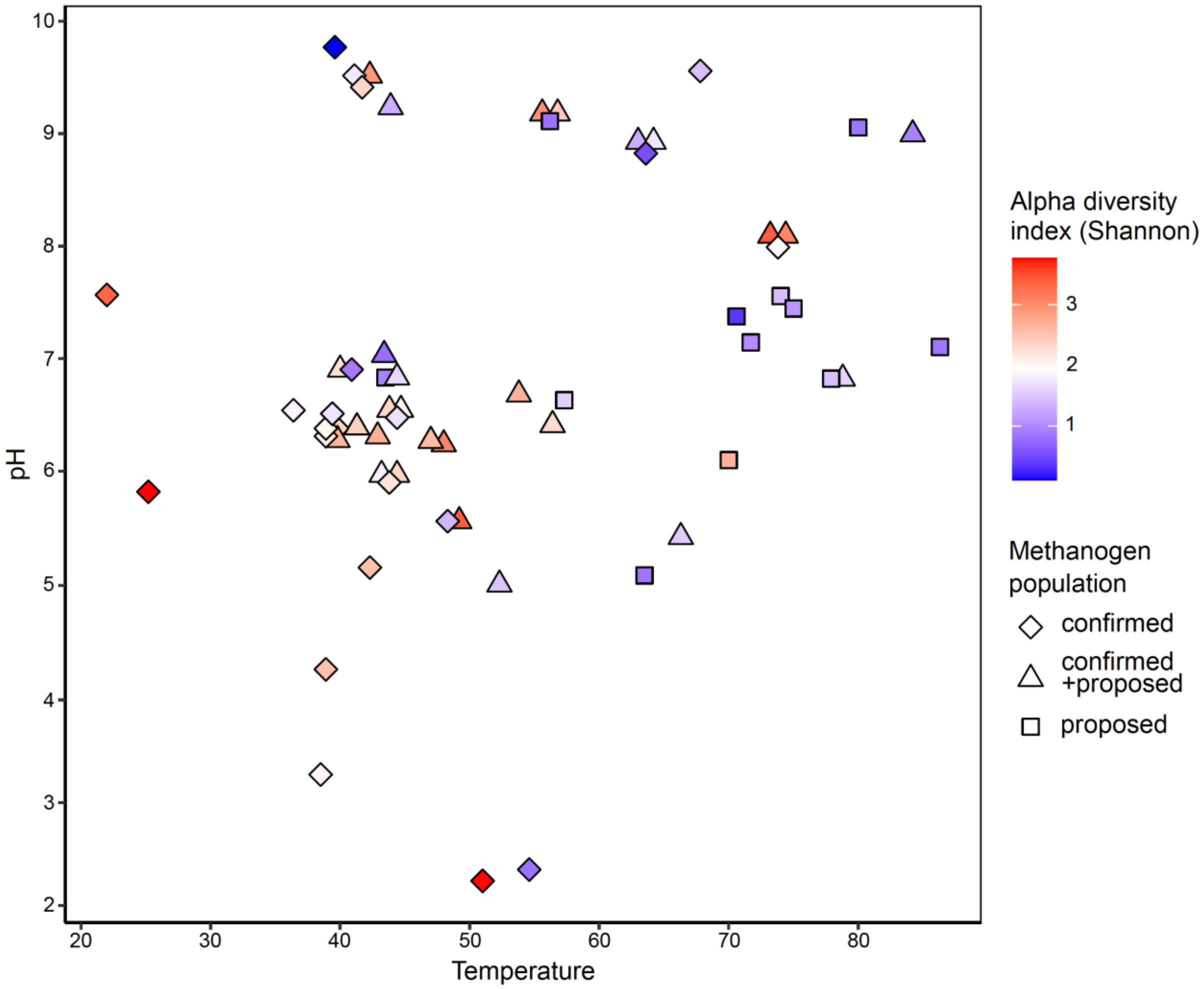


**SI Figure 1.** Alpha diversity of *mcrA* gene ASVs across temperature and pH. Most samples were retrieved from geothermal features with circumneutral pH and moderate to elevated temperatures (35-80ºC). *mcrA* alpha diversity (Shannon index) indicates a trend towards decreased richness at elevated temperatures. Mcr-encoding communities tend to be primarily composed of confirmed methanogens at moderate temperatures and proposed methanogens or methane/alkane oxidizing archaea at elevated temperatures.

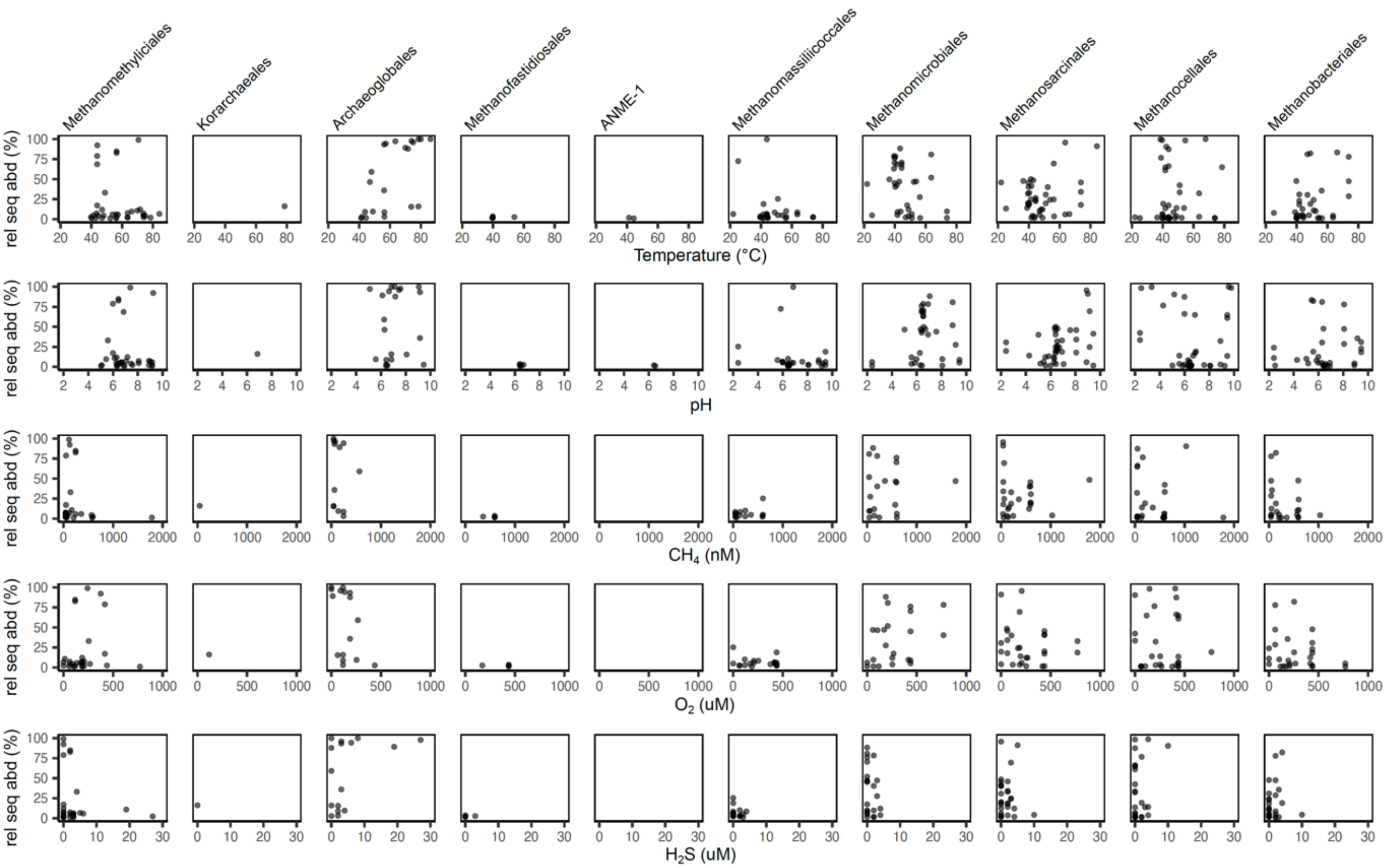


**SI Figure 2**. Distribution of Mcr-encoding archaea across physicochemical regimes. Mcr-encoding lineages detected via *mcrA* gene amplicon sequencing with relative abundances >1%. Aqueous $CH_4$, $O_2$, and $H_2S$ data not available for all samples. Detection limit for $O_2$ and $H_2S$ was 13 μM and 2 μM, respectively.

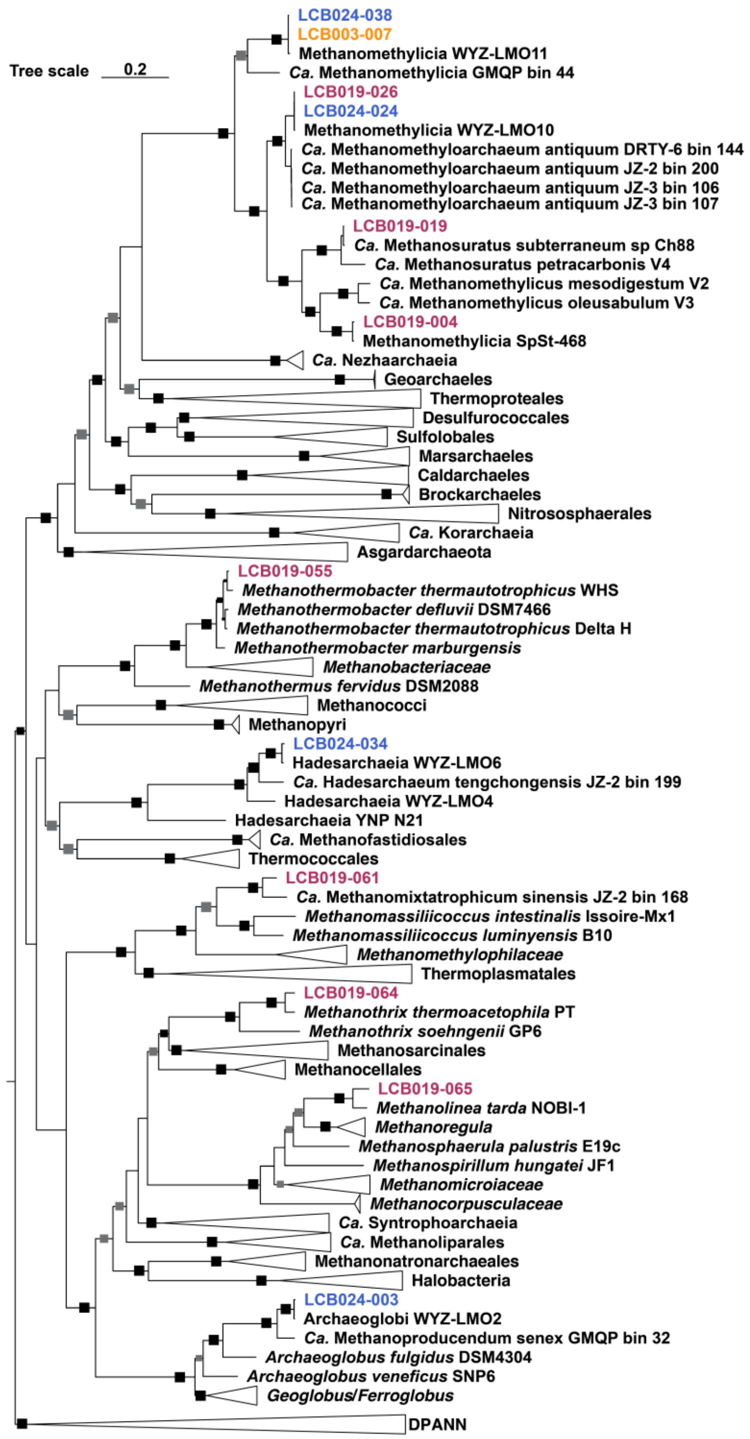


**SI Figure 3.** Phylogenetic affiliation of Mcr-encoding MAGs. Expanded maximum-likelihood tree, inferred with IQtree and the best-fit LG+C60+F+G model, from the concatenated alignment of 18 conserved arCOGs (SI Table 4). Squares indicate ultrafast bootstrap values of 100% (black) and 95-99% (gray).

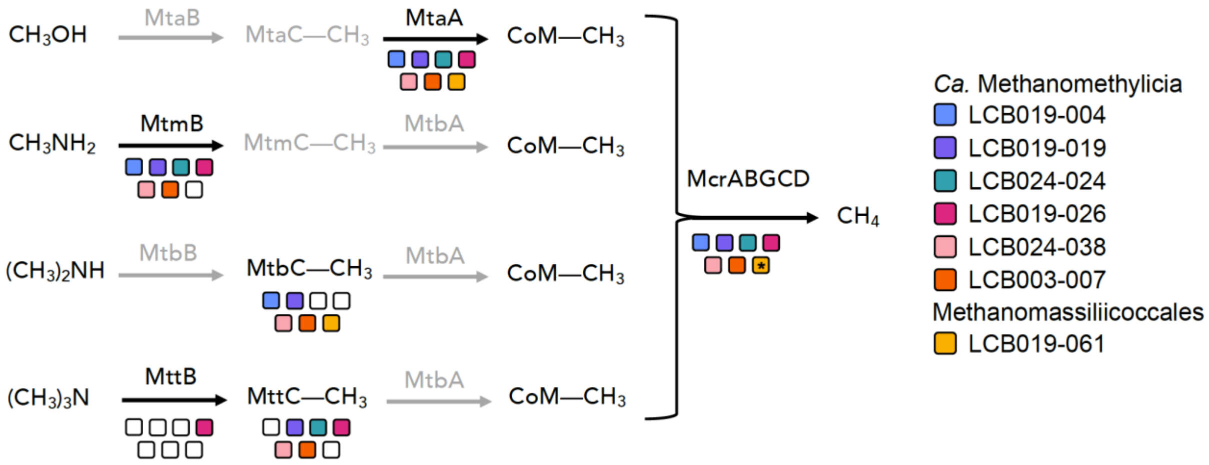


**SI Figure 4.** Proposed pathways for methylotrophic methanogenesis in MAGs from this study. Colors indicate individual MAGs affiliated with *Ca.* Methanomethylicia and Methanomassiliicoccales. Filled squares indicate methyltransferases and corrinoid proteins detected in the MAG. Shaded in gray are methyltransferases and corrinoid proteins not detected in any MAG. * denotes absence of McrG in LCB019-061. See SI Data 3 for details on protein abbreviations.

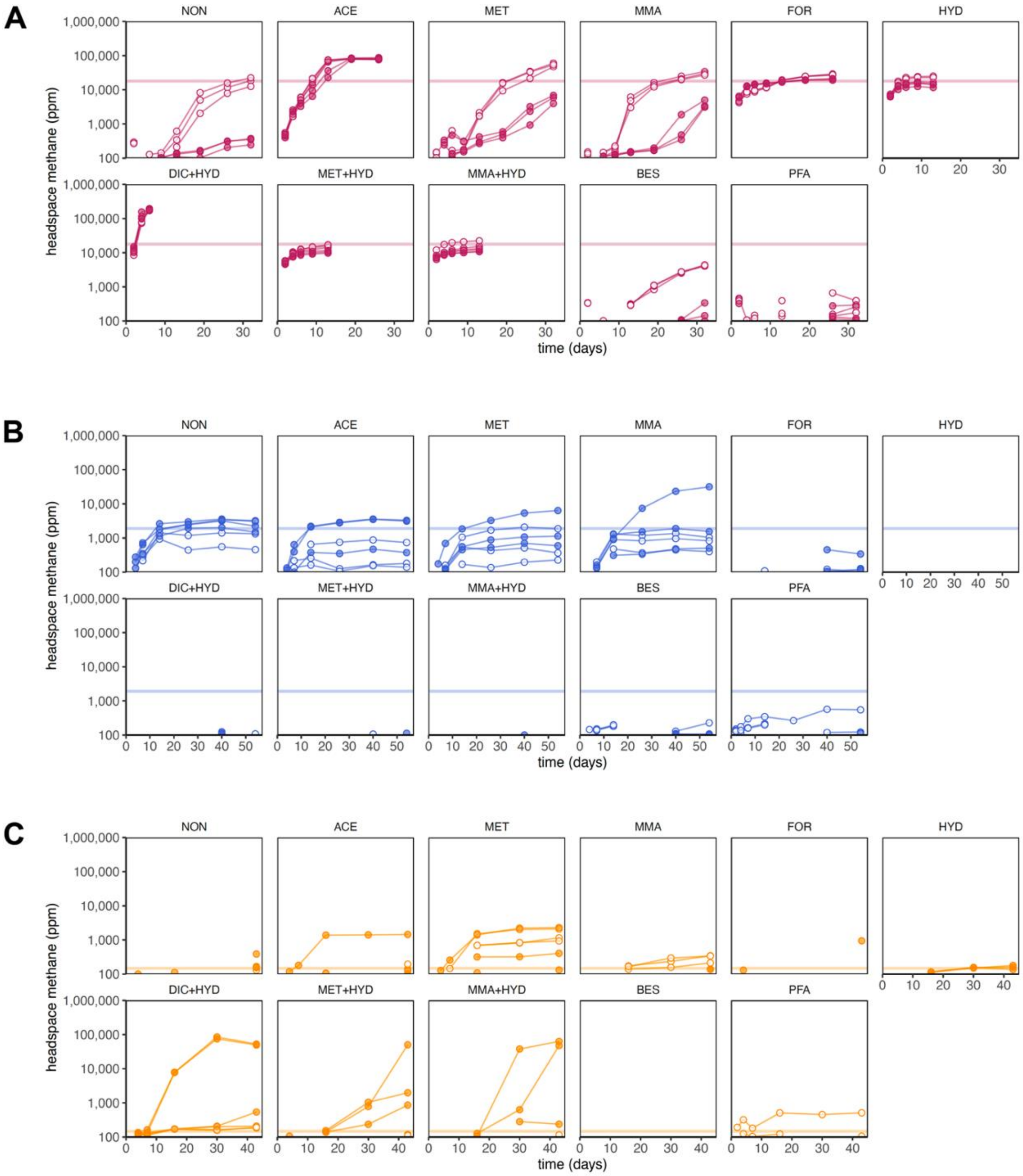


**SI Figure 5.** Development of methane concentrations in the headspace of mesocosms. (A) LCB019, (B) LCB024 and (C) LCB003. Open circles, without antibiotics; filled circles, with antibiotics. Horizontal line: average maximum methane produced in controls without substrate amendment and without antibiotics. Detection limit: 100 ppm. Abbreviations: NON, no substrate amendment; FOR, formate; ACE, acetate; MET, methanol; MMA, monomethylamine; HYD, hydrogen; MMA+HYD, monomethylamine plus hydrogen; MET+HYD, methanol plus hydrogen; DIC+HYD, dissolved inorganic carbon ($HCO_3^-$+$CO_2$) plus hydrogen; BES, bromoethanesulfonate (methanogenesis inhibitor); PFA, paraformaldehyde (killed control).

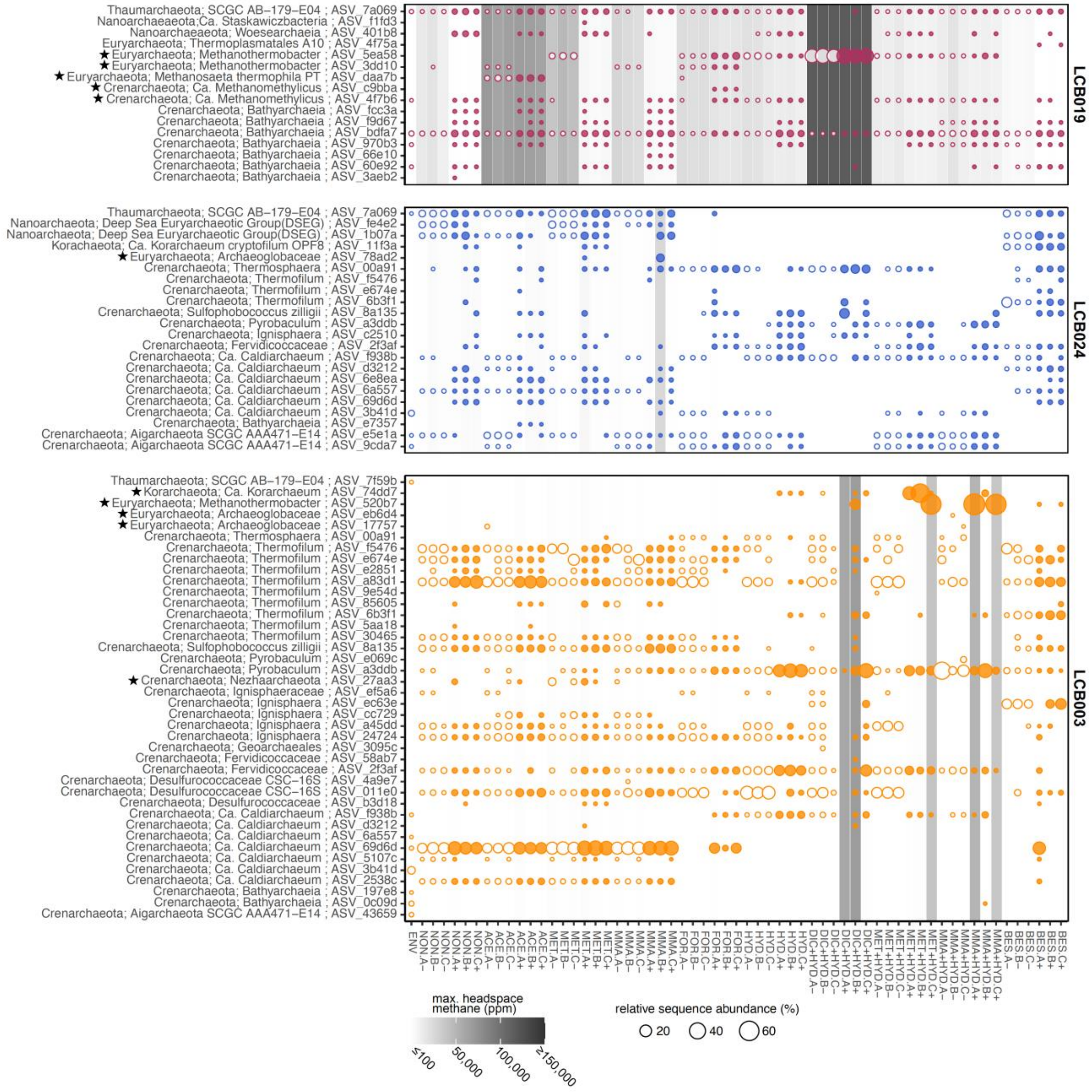


**SI Figure 6.** Relative sequence abundance of archaeal ASVs detected at >1% in mesocosms. Stars indicate ASVs identified as affiliated with Mcr-encoding archaea discussed in this study. Open circles, without antibiotics; filled circles, with antibiotics. Color coding by site: orange, LCB003, magenta, LCB019, blue, LCB024. Circle size proportional to relative sequence abundance. Gray shading indicates maximum methane concentration detected in mesocosm headspace. Abbreviations: ENV, environmental sample; NON, no substrate amendment; FOR, formate; ACE, acetate; MET, methanol; MMA, monomethylamine; HYD, hydrogen; MMA+HYD, monomethylamine plus hydrogen; MET+HYD, methanol plus hydrogen; DIC+HYD, dissolved inorganic carbon ($HCO_3^-$+$CO_2$) plus hydrogen; BES, bromoethanesulfonate (methanogenesis inhibitor); PFA, paraformaldehyde (killed control).

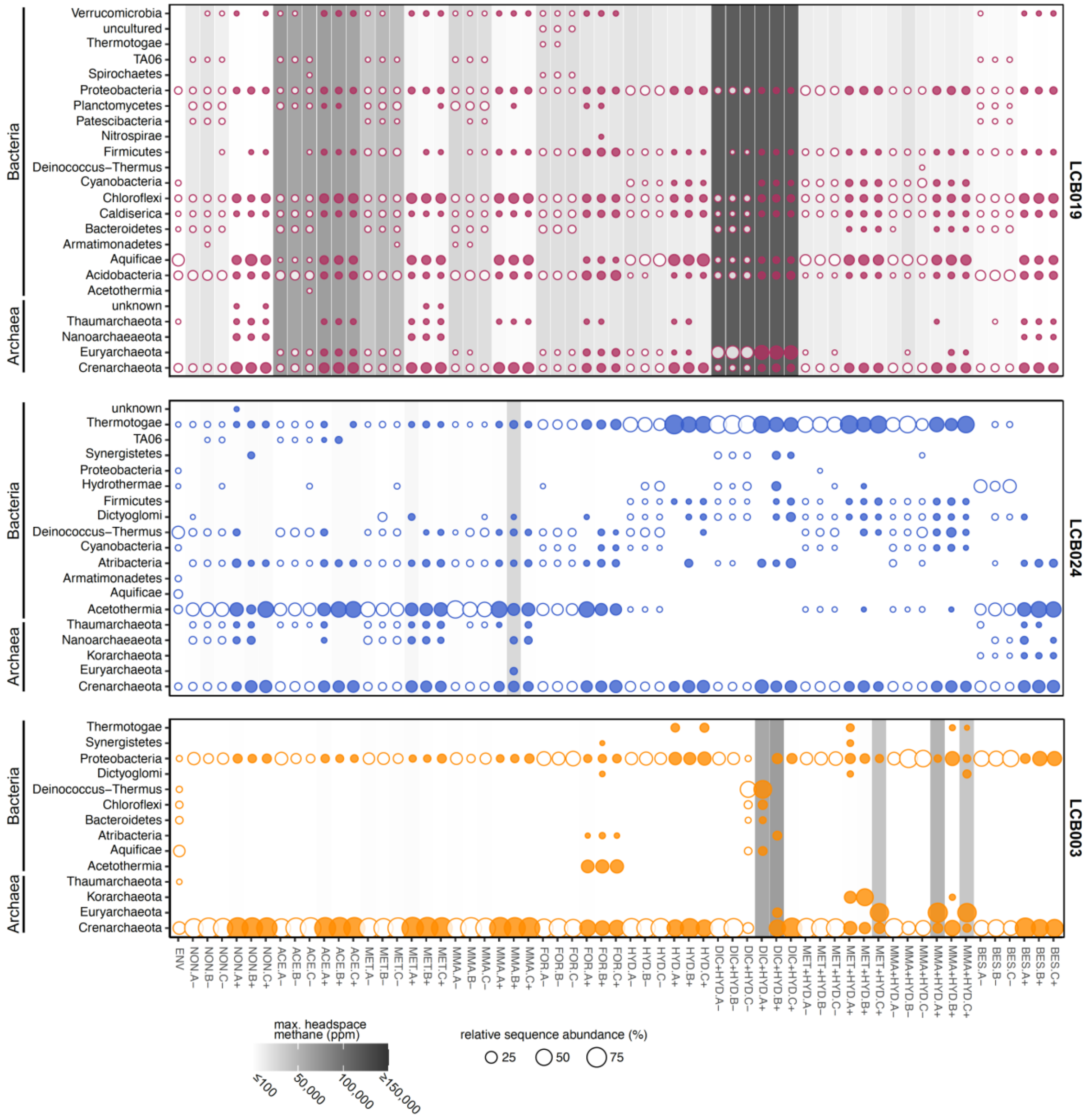


**SI Figure 7.** Relative sequence abundance of phyla detected at >3% in mesocosms. Open circles, without antibiotics; filled circles, with antibiotics. Color coding by site: orange, LCB003, magenta, LCB019, blue, LCB024. Circle size proportional to relative sequence abundance. Gray shading indicates maximum methane concentration detected in mesocosm headspace. Abbreviations: ENV, environmental sample; NON, no substrate amendment; FOR, formate; ACE, acetate; MET, methanol; MMA, monomethylamine; HYD, hydrogen; MMA+HYD, monomethylamine plus hydrogen; MET+HYD, methanol plus hydrogen; DIC+HYD, dissolved inorganic carbon ($HCO_3^-$ +$CO_2$) plus hydrogen; BES, bromoethanesulfonate (methanogenesis inhibitor); PFA, paraformaldehyde (killed control).

## Extended Materials and Methods

### Physicochemical measurements, aqueous geochemistry, and elemental analysis.

Prior to removing any sediment or microbial mat material from geothermal features, pH and temperature were recorded using a thermocouple (Fluke 52 II; Everett, WA) and a portable pH meter (ThermoFisher Orion Star A329 with electrode 8107UWMMD, Accumet AP63 with electrode AP50A, Accumet AP71 with electrode AP55, Waltham, MA), and geothermal water was collected for analysis of aqueous geochemistry and elements. Water samples were filtered through a pre-rinsed 0.22 µm PES syringe filter directly into sterile screw-cap plastic tubes and carbon-free glass vials sealed headspace-free. Blanks were prepared with ultrapure water (Barnstead, ThermoFisher, 18.2 MΩ). Samples and blanks were stored at 4 ˚C until analysis by the Environmental Analytical Lab (Montana State University). An aliquot was acidified with trace-metal grade $HNO_3$ (20%) to a final concentration of 5% and analyzed using inductively coupled plasma optical emission spectroscopy (ICP-OES) (SpectroBLUE, Kleve, Germany) for total dissolved Ag, Al, As, Ba, Be, Bi, Ca, Cd, Co, Cr, Cu, Fe, Mg, Mn, Mo, Ni, Pb, Sb, Se, Sr, Ti, Tl, V, Zn, B, K, Li, Na, S, Si. An unacidified aliquot was analyzed for $SO_4^{2-}$, $Cl^-$, and $F^-$ using a Dionex (Sunnyvale, CA, USA) ICS-2100 ion chromatograph with AS18 column and $NO_2^-$, $PO_4^{3-}$, and $NH_4^+$ using a segmented flow analyzer (Seal Analytical, Southampton, UK). Samples collected in carbon-free glass vials were analyzed for total carbon (TC), inorganic carbon (IC), non-purgeable organic carbon (NPOC), and total nitrogen (TN) using a carbon analyzer (Shimadzu, Kyoto, Japan).

Dissolved oxygen (DO) was determined using a closed-headspace modification of the Winkler protocol (1). Briefly, 60 mL of geothermal water were collected with a 60 mL syringe that was immediately capped with a rubber septum to prevent gas exchange with the atmosphere. Needles and syringes were used to inject 0.4 mL of 2.15 M $MnSO_4$ and 0.4 mL alkali-iodide-azide solution (12 M NaOH, 0.869 M KI, 0.15 M $NaN_3$). The sample was thoroughly mixed by inversion and the formed floc was allowed to settle for 3-5 minutes. This mixing process was repeated twice before 0.4 mL concentrated (18 M) $H_2SO_4$ were added, and the floc completely dissolved by mixing. 30 mL of this mixture were titrated to colorlessness with 10.1 mM sodium thiosulfate to determine DO.

Total dissolved sulfide ($H_2S(aq)$) was measured using the amine-sulfuric acid method (2). For this, 7.5 mL of geothermal water were collected with a 10 mL syringe and added into triplicate

glass tubes pre-filled with 0.5 mL of amine sulfuric acid reagent (Ricca Chemical). Approximately 0.15 mL (4 drops) of 15 M $FeCl_3$ were immediately added and the sample was mixed by slowly inverting the test tube once. After 3-5 minutes, 1.6 mL of 3.8 M $(NH_4)_2PO_4$ were added and the sample was stored in the dark until absorbance was measured at 664 nm using a BioMate 3S spectrophotometer or Ocean Optics USB2000 spectrophotometer within 24 hours of sample collection.

The ferrozine assay was used to measure total and ferrous iron (Fe, Fe(II)) (3). Briefly, 5 mL of geothermal water were filtered through a 0.22 µm pore size PES syringe filter into three replicate tubes. 250 µL of a 10% w/v hydroxylamine solution were added for the Fe(II) assay before 250 µL of 4.9 mM ferrozine reagent and 625 µL ammonium-acetate buffer (3) were added for both Fe total and Fe(II) assays. After dilution to 12.5 mL with distilled water, samples were stored in the dark until absorbance was measured at 562 nm using a BioMate 3S spectrophotometer or Ocean Optics USB2000 spectrophotometer within 24 hours of sample collection.

To determine dissolved methane ($CH_4$(aq)), geothermal water was collected using a peristaltic pump with an in-line 142 mm diameter, 0.2 µm pore size hydrophilic polycarbonate membrane filter (Millipore). 160 mL serum bottles were purged with three pour volumes of geothermal water, capped without headspace using butyl rubber stoppers, secured with aluminum rings, and stored upside-down at 4 ºC. Prior to the analysis of dissolved gases, samples were weighed before and after 30 mL of liquid was removed, headspace replaced with ambient air, and incubated on a shaker at 60 rpm for two hours at room temperature. From the headspace, 1 mL was withdrawn using a gas tight syringe and manually injected into an SRI Multiple Gas Analyzer #5 gas chromatograph equipped with a Haysep D column (6 foot), a thermal conductivity detector, and a flame ionization detector with a methanizer (carrier gas: $N_2$ at 13 psi, 50ºC). Methane and mixed gas ($CH_4$, $CO_2$, CO, $H_2$) standards (100 ppm, 10,000 ppm) were measured regularly. Concentrations of dissolved gases were calculated using Henry's law and solubility equations (2, 4).

### ***mcrA*** **and 16S rRNA gene amplification and amplicon sequencing.**

*mcrA* genes were amplified from DNA extracts of environmental samples and 16S rRNA genes were amplified from DNA extracts of mesocosm samples including the initial environmental material (environmental sample). DNA extracts from 500 µL sterile nuclease-free water were included as negative controls. DNA extracts were quantified using the Qubit high sensitivity assay

(Invitrogen, Carlsbad, CA). Adapter sequences added to the gene-specific primers facilitated the preparation of Illumina amplicon libraries for sequencing as previously described (5).

The *mcrA* gene was amplified using the primer set mlas-mod-F/mcrA-rev-R (6). The PCR volume was 25 µL and consisted of 10 µL Invitrogen Platinum Taq II 2X Master Mix, 2 µL mlas-mod-F primer (10 µM; final 0.8 µM), 2 µL mcrA-rev-R primer (10 µM; final 0.8 µM), 10 µL nuclease-free water, and 1 µL of template DNA (5 ng/µL). Thermocycler conditions for amplification were: 94ºC for 4 min, followed by five touchdown cycles of 94ºC for 30 sec, 60–1 ºC for 45 sec, and 72ºC for 30 sec, followed 30 cycles of 94 ºC for 30 sec, 55 ºC for 30 sec and 72 ºC for 30 sec, and a final elongation step at 72 ºC for 10 min (6).

Archaeal and bacterial 16S rRNA genes were amplified following the Earth Microbiome protocol with the updated primer set 515F and 806R (7-9). The PCR volume was 25 µL and consisted of 10 µL Invitrogen Platinum Taq II 2X Master Mix, 0.5 µL 515F primer (10 µM; final: 0.2 µM), 0.5 µL 806R primer (10 µM; final: 0.2 µM), 9 µL nuclease-free water, and 5 µL of template DNA (1 ng/µL). The thermocycler conditions were: 94 °C for 3 min followed by 28 cycles of 94 °C for 45 sec, 50 °C for 60 sec, and 72 °C for 90 sec before a final elongation step at 72 °C for 10 min.

A negative control with nuclease-free water as template was included in each PCR set. PCR products were checked for the expected length on a 1% agarose gel. *mcrA* and 16S rRNA gene amplicons were purified using AMPure XR beads (Beckman Coulter, Pasadena, CA) following the manufacturer's protocol with a final elution volume of 40 µL nuclease-free water before a second PCR was performed to attach dual barcode indices and sequence adapters (Illumina). This PCR was performed in a 25 µL final volume with 5 µL purified amplicons 12.5 µL Invitrogen Platinum Taq II 2X Master Mix, 2.5 µL i5 primer (10µM; final: 0.25 µM), 2.5 µL i7 primer (10 µM; final: 0.25 µM), and 2.5 µL nuclease-free water. The PCR conditions were as follows: 95 °C for 3 min followed by 8 cycles of 95 °C for 30 sec, 55 °C for 30 sec, and 72 °C for 30 sec, followed by a final elongation step at 72 °C for 5 min. The amplicons were purified using AMPure XR beads and were quantified in triplicate reactions using the Quant-iT Picogreen dsDNA Assay (Invitrogen, Waltham, MA) and a Biotek Synergy H1 Hybrid microplate reader following manufacturers guidelines. Samples were pooled at 10 ng DNA each, concentrated using a QIAquick PCR purification spin column kit (Qiagen, Hilden, Germany) following the

manufacturers protocol, and quantified with the Qubit high sensitivity assay (Invitrogen, Waltham, MA).

**Metagenome sequencing, read processing, assembly, and annotation.**

Truseq libraries were prepared at the JGI using low (10ng, LCB003.1) or regular (100ng, LCB019.1, LCB024.1) input quantities of DNA and were sequenced on the Illumina NovaSeq platform using the NovaSeq XP V1 reagent kits, S4 flow cell, following a 2 x 150 bp indexed run recipe. Raw metagenomic reads were processed according to the standard workflow employed by the JGI. Briefly, BBDuk (version 38.08; https://bbtool.jgi.doe.gov) was used to remove contaminants, trim reads that contained adapter sequence, right quality trim reads where quality drops to zero and remove reads that contained four or more 'N' bases, had an average quality score across the read less than 3 or had a minimum length <= 51 bp or 33% of the full read length. Reads mapped with BBMap (v37.78) to masked human, cat, dog, and mouse references at 93% identity were removed as were reads aligned to common microbial contaminants (JGI SOP 1077)(10). Trimmed and screened paired-end reads were error corrected with bfc (v. r181) with options: -1, -s 10g, -k 21, t 10 (Li, 2015) and then assembled using SPAdes 3.11.1 (11) with options -m 2000, -k 33,55,77,99,127 -meta. Coverage information was generated using BBMap by mapping the entire filtered reads dataset against the final assembly. Annotation of the final assembly was performed with JGI's Integrated Microbial Genomes & Microbiomes Expert Review (IMG/M-ER) pipeline v7 (12). Assembled scaffolds ≥2,000 bp were binned with six implementations of four different programs including, Maxbin v2.2.4 (13), Concoct v1.0.0 (14), Metabat v2.12.1 (with and without coverage) (15), and Autometa v1 (with bacterial and archaeal modes, and Machine Learning step, but omitting the taxonomy assignment step) (16). Bins generated from each method were dereplicated, aggregated, and scored with DAS_Tool (17).

## Extended Discussion

### Energy conservation mechanisms of Mcr-encoding archaea

Both LCB019-065 (*Methanolinea*) and LCB019-055 (*Methanothermobacter*) encode heterodisulfide reductase (HdrABC) and either $F_{420}$-non-reducing hydrogenase (MvhADG; LCB019-055) or $F_{420}$-reducing hydrogenase (FrhAG) and MvhD (LCB019-065) allowing for two distinct mechanisms of heterodisulfide (CoM-S-S-CoB) reduction. In LCB019-055, heterodisulfide reduction could be mediated by an electron bifurcating HdrABC/MvhADG complex as is common for other hydrogenotrophic methanogens (18). Conversely, in LCB019-065, HdrABC could form an electron bifurcating complex with FrhAG and MvhD to reduce heterodisulfide as described in some members of the Methanomicrobiales (18-20).

LCB019-064 (*Methanothrix*) encodes HdrDE and a Fpo-like complex (*fpoABCDHIJKLMN*, lacking *fpoFO* for binding and oxidizing $F_{420}$), which would allow for heterodisulfide reduction by HdrDE with electrons supplied by the Fpo-like complex. The [NiFe] hydrogenase Frh, energy-converting hydrogenase (Ech), $Na^+$-translocating ferredoxin:$NAD^+$ oxidoreductase (Rnf), and methanophenazine-reducing hydrogenase (Vho) were not encoded. This suggests that energy conservation in LCB019-064 may proceed as proposed for *Methanothrix thermophila* (21) and differs from the energy conservation mechanisms of other aceticlastic methanogens within the genus *Methanosarcina*.

Methanomassiliicoccales MAG LCB019-061 encodes HdrABC, MvhADG, and a partial Fpo-like complex (21), which is consistent with the energy conservation complexes found in $H_2$-dependent methylotrophic methanogens (22). An electron bifurcating HdrABC/MvhADG complex (23, 24), could couple the oxidation of $H_2$ with the reduction of heterodisulfide and ferredoxin. Interestingly, multiple copies of the *hdrA* (x14), *hdrB* (x4), and *hdrC* (x4) genes were identified in LCB019-061 while *hdrD* was not detected (SI Data 3). The oxidation of reduced ferredoxin by the membrane bound Fpo-like complex would generate a proton gradient for ATP synthesis via a V/A-type ATP synthase.

The energy conservation complexes encoded in the six *Ca*. Methanomethylicia MAGs (LCB003-007, LCB019-004, LCB019-019, LCB019-026, LCB024-024, and LCB024-038) were similar to those in related MAGs (26). Genes encoding complexes involved in establishing a proton gradient, including the Fpo-like complex (21) and an energy-converting hydrogenase B (Ehb) complex, were detected in all six MAGs (27). The generated proton gradient would drive ATP

synthesis via a V/A-type ATP synthase, encoded in all MAGs. Previously proposed mechanisms of heterodisulfide reduction encoded in the six *Ca*. Methanomethylicia MAGs include reduction via an interaction between HdrD and the Fpo-like complex (25), a Fpo-HdrD-GlcD complex (26), or energy-converting hydrogenase D (Ehd) formed by a membrane-bound [NiFe] hydrogenase and HdrBC (28). All six MAGs contained 1-3 copies of *hdrD* and *glcD*, however the Ehd complex was only encoded in four MAGs (LCB003-007, LCB019-026, LCB024-038, and LCB019-019 (partially)).

LCB024-003 (Archaeoglobi) encodes two mechanisms for coenzymes M and B regeneration. LCB024-003 contains *mvhADG*, *hdrD*, and three copies of *hdrA* (one upstream of *mvhADG). hdrBC* were not detected; however, HdrD has been proposed to substitute for HdrBC in heterodisulfide reduction in other Mcr-encoding Archaeoglobi MAGs (29, 30). LCB024-003 also encodes a fused HdrDE and a membrane-bound $F_{420}H_2$:quinone oxidoreductase (Fqo) complex. In Archaeoglobi isolates, the Fqo complex has been shown to reduce menaquinone instead of methanophenazine (31), which is characteristic of methanogens containing an Fpo complex (32). HdrDE contains two cysteine-rich domains (CCG) and is homologous to the membrane bound complex DsrMK, which is inferred to reduce a cytoplasmic cysteine disulfide (Cys-S-S-Cys) in DsrC during dissimilatory sulfate reduction (33). Similarly, HdrDE could couple the periplasmic oxidation of menaquinol, generated from Fqo, to the cytoplasmic reduction of the disulfide bond in CoM-S-S-CoB. A replacement of HdrDE for DsrMK has been shown in *Archaeoglobus fulgidus* VC16 (34) and has been proposed for other Mcr-encoding Archaeoglobi MAGs (30).

## References


1. Macur RE, Jay ZJ, Taylor WP, Kozubal MA, Kocar BD, Inskeep WP. Microbial community structure and sulfur biogeochemistry in mildly-acidic sulfidic geothermal springs in Yellowstone National Park. Geobiology. 2013;11(1):86-99.
2. Inskeep WP, Ackerman GG, Taylor WP, Kozubal MA, Korf S, Macur RE. On the energetics of chemolithotrophy in nonequilibrium systems: case studies of geothermal springs in Yellowstone National Park. Geobiology. 2005:297-317.
3. To TB, Nordstrom DK, Cunningham KM, Ball JW, McCleskey RB. New Method for the Direct Determination of Dissolved Fe(III) Concentration in Acid Mine Waters. Environmental Science & Technology. 1999;33(5):807-13.
4. Amend JP, Shock EL. Energetics of overall metabolic reactions of thermophilic and hyperthermophilic Archaea and bacteria. FEMS Microbiol Rev. 2001;25(2):175-243.
5. Reichart NJ, Jay ZJ, Krukenberg V, Parker AE, Spietz RL, Hatzenpichler R. Activity-based cell sorting reveals responses of uncultured archaea and bacteria to substrate amendment. ISME J. 2020;14(11):2851-61.
6. Angel R, Claus P, Conrad R. Methanogenic archaea are globally ubiquitous in aerated soils and become active under wet anoxic conditions. The ISME Journal. 2012;6(4):847-62.
7. Thompson LR, Sanders JG, McDonald D, Amir A, Ladau J, Locey KJ, et al. A communal catalogue reveals Earth's multiscale microbial diversity. Nature. 2017;551(7681):457-63.
8. Parada AE, Needham DM, Fuhrman JA. Every base matters: assessing small subunit rRNA primers for marine microbiomes with mock communities, time series and global field samples. Environ Microbiol. 2016;18(5):1403-14.
9. Apprill A, McNally S, Parsons R, Weber L. Minor revision to V4 region SSU rRNA 806R gene primer greatly increases detection of SAR11 bacterioplankton. Aquatic microbial ecology : international journal. 2015;75(2):129-37.
10. Huntemann M, Ivanova NN, Mavromatis K, Tripp HJ, Paez-Espino D, Palaniappan K, et al. The standard operating procedure of the DOE-JGI Microbial Genome Annotation Pipeline (MGAP v.4). Stand Genomic Sci. 2015;10:86.
11. Nurk S, Meleshko D, Korobeynikov A, Pevzner PA. metaSPAdes: a new versatile metagenomic assembler. Genome Res. 2017;27(5):824-34.

12. Chen IA, Markowitz VM, Chu K, Palaniappan K, Szeto E, Pillay M, et al. IMG/M: integrated genome and metagenome comparative data analysis system. Nucleic Acids Res. 2017;45(D1):D507-D16.
13. Wu Y-W, Tang Y-H, Tringe SG, Simmons BA, Singer SW. MaxBin: an automated binning method to recover individual genomes from metagenomes using an expectation-maximization algorithm. Microbiome. 2014;2(1):26.
14. Alneberg J, Bjarnason BS, de Bruijn I, Schirmer M, Quick J, Ijaz UZ, et al. Binning metagenomic contigs by coverage and composition. Nat Methods. 2014;11(11):1144-6.
15. Kang DD, Froula J, Egan R, Wang Z. MetaBAT, an efficient tool for accurately reconstructing single genomes from complex microbial communities. PeerJ. 2015;3:e1165.
16. Miller IJ, Rees ER, Ross J, Miller I, Baxa J, Lopera J, et al. Autometa: automated extraction of microbial genomes from individual shotgun metagenomes. Nucleic Acids Res. 2019;47(10):e57.
17. Sieber CMK, Probst AJ, Sharrar A, Thomas BC, Hess M, Tringe SG, et al. Recovery of genomes from metagenomes via a dereplication, aggregation and scoring strategy. Nat Microbiol. 2018;3(7):836-43.
18. Thauer RK, Kaster AK, Goenrich M, Schick M, Hiromoto T, Shima S. Hydrogenases from methanogenic archaea, nickel, a novel cofactor, and H2 storage. Annu Rev Biochem. 2010;79:507-36.
19. Thauer RK, Kaster AK, Seedorf H, Buckel W, Hedderich R. Methanogenic archaea: ecologically relevant differences in energy conservation. Nat Rev Microbiol. 2008;6(8):579-91.
20. Anderson I, Ulrich LE, Lupa B, Susanti D, Porat I, Hooper SD, et al. Genomic characterization of methanomicrobiales reveals three classes of methanogens. PLoS One. 2009;4(6):e5797.
21. Welte C, Deppenmeier U. Membrane-bound electron transport in Methanosaeta thermophila. J Bacteriol. 2011;193(11):2868-70.
22. Speth DR, Orphan VJ. Metabolic marker gene mining provides insight in global mcrA diversity and, coupled with targeted genome reconstruction, sheds further light on metabolic potential of the Methanomassiliicoccales. PeerJ. 2018;6:e5614.

23. Kroninger L, Berger S, Welte C, Deppenmeier U. Evidence for the involvement of two heterodisulfide reductases in the energy-conserving system of Methanomassiliicoccus luminyensis. FEBS J. 2016;283(3):472-83.
24. Wagner T, Koch J, Ermler U, Shima S. Methanogenic heterodisulfide reductase (HdrABC-MvhAGD) uses two noncubane [4Fe-4S] clusters for reduction. Science. 2017;357(6352):699-703.
25. Lang K, Schuldes J, Klingl A, Poehlein A, Daniel R, Brunea A. New mode of energy metabolism in the seventh order of methanogens as revealed by comparative genome analysis of "Candidatus methanoplasma termitum". Appl Environ Microbiol. 2015;81(4):1338-52.
26. Vanwonterghem I, Evans PN, Parks DH, Jensen PD, Woodcroft BJ, Hugenholtz P, et al. Methylotrophic methanogenesis discovered in the archaeal phylum Verstraetearchaeota. Nat Microbiol. 2016;1:16170.
27. Nobu MK, Narihiro T, Kuroda K, Mei R, Liu WT. Chasing the elusive Euryarchaeota class WSA2: genomes reveal a uniquely fastidious methyl-reducing methanogen. ISME J. 2016;10(10):2478-87.
28. Borrel G, Adam PS, McKay LJ, Chen LX, Sierra-Garcia IN, Sieber CMK, et al. Wide diversity of methane and short-chain alkane metabolisms in uncultured archaea. Nat Microbiol. 2019;4(4):603-13.
29. Hedderich R, Hamann N, Bennati M. Heterodisulfide reductase from methanogenic archaea: a new catalytic role for an iron-sulfur cluster. Biol Chem. 2005;386(10):961-70.
30. Liu YF, Chen J, Zaramela LS, Wang LY, Mbadinga SM, Hou ZW, et al. Genomic and Transcriptomic Evidence Supports Methane Metabolism in Archaeoglobi. mSystems. 2020;5(2).
31. Bruggemann H, Falinski F, Deppenmeier U. Structure of the F420H2:quinone oxidoreductase of Archaeoglobus fulgidus identification and overproduction of the F420H2-oxidizing subunit. Eur J Biochem. 2000;267(18):5810-4.
32. Ferry JG. How to make a living by exhaling methane. Annu Rev Microbiol. 2010;64:453-73.

33. Pereira IA, Ramos AR, Grein F, Marques MC, da Silva SM, Venceslau SS. A comparative genomic analysis of energy metabolism in sulfate reducing bacteria and archaea. Front Microbiol. 2011;2:69.

34. Hocking WP, Roalkvam I, Magnussen C, Stokke R, Steen IH. Assessment of the Carbon Monoxide Metabolism of the Hyperthermophilic Sulfate-Reducing Archaeon Archaeoglobus fulgidus VC-16 by Comparative Transcriptome Analyses. Archaea. 2015;2015:235384.
